# EquiCity game: a mathematical serious game for participatory design of spatial configurations

**DOI:** 10.1038/s41598-024-61093-4

**Published:** 2024-05-13

**Authors:** Pirouz Nourian, Shervin Azadi, Nan Bai, Bruno de Andrade, Nour Abu Zaid, Samaneh Rezvani, Ana Pereira Roders

**Affiliations:** 1 Department of Planning and Geoinformation Management, University of Twente, 7522 NH Enschede, Netherlands; 2 Department of Built Environment, Eindhoven University of Technology, 5612 AZ Eindhoven, Netherlands; 3 Department of Engineering and Technology, Delft University of Technology, 2628 BL Delft, Netherlands; 4 Department of Architecture and Multimedia Gallaecia, University Portucalense, 4200-072 Porto, Portugal; 5 Forensic Architecture Research Group, Goldsmiths University of London, London, SE14 6NW UK; 6 Department of Research and Development, DEMO Consultants B.V., 2628 XJ Delft, Netherlands

**Keywords:** Civil engineering, Sustainability

## Abstract

We propose a mathematical framework for developing social-choice games that are designed to mediate decision-making processes for city planning, urban area redevelopment, and architectural configuration of urban housing complexes. The proposed framework features a digital serious gaming approach for participatory design to support transparency and inclusion in the process of decision-making and ensure an equitable balance of sustainable development goals in spatial design outcomes. The mathematical process consists of a Markovian design machine for balancing the design decisions of actors, a massing configurator equipped with fuzzy logic and multi-criteria decision analysis, algebraic graph-theoretical accessibility evaluators, and automated solar-climatic evaluators using geospatial computational geometry. We demonstrate the effectiveness of the framework by implementing a multi-player online game that facilitates a participatory decision-making workshop for forming multi-functional building complexes by providing a generative configurator equipped with automated appraisal/scoring mechanisms for revealing the aggregate impact of alternatives. The EquiCity game empowers a group of decision-makers to reach a fair consensual spatial design by mathematically simulating many rounds of reasonable trade-offs between their decisions, with different levels of interest or control over various types of investments. The novelty of the framework is in its capability to encompass decision-making about the most idiosyncratic aspects of a site related to its heritage status and cultural significance to the most generic aspects such as balancing access to sunlight for the site while respecting ‘the right to sunlight’ of the neighbours of the site, ensuring coherence of the entire configuration with regards to a network of desired closeness ratings, the satisfaction of a programme of requirements, and intricately balancing individual development goals in conjunction with communal goals and environmental design codes.

## Introduction

The problem framed in this paper generally pertains to the challenge of reaching satisfactory decisions with respect to multiple criteria by a group of actors on the allocation of various resources^1^ into some kind of an investment portfolio, subject to some budget constraints, and quality criteria, with different value systems, possibly conflicting individual goals and various uneven levels of interest and control on the resources and investments. While this general picture is recognisable in multiple forms such as organizational decision-making and planning in general, this paper addresses such resource allocation problems in the context of urban architecture and area development, proposing a methodology for facilitating consensus-building and participatory design/decision-making to achieve ‘consensual satisfaction of multiple criteria’. The generality of such resource-allocation problems on the one hand and the specific challenges arising out of the spatial complexity of the participatory urban-architectural design problem, on the other hand, motivated our mathematical research for devising a game engine for equitable decision-making in the context of spatial developments.

In short, this research provided an opportunity for testing the potential of games as ’play & score’ mechanisms for facilitating equitable decision-making in dealing with complex urban development problems and their constituent public or community-owned resources. See other examples such as^2,3^, a classification of Simulation Games^4^, two comprehensive books by Sanoff respectively on participatory planning and design games,^5,6^, a reference on developing games for participatory stakeholder analysis^7^, a review on city-making games^8^, an interesting application of games in Transport Planning, explaining the value of the communicative-rational approach to planning in comparison to the technical-rational approach^9^, the pioneering book of Epstein on Generative Social Science for its analysis of consensus as equilibrium in multi-player games^10^, an introduction to Planning Support Systems^11^, a game-theoretical treatise on evolution around equilibrium in multi-player games^12^, a classical book on the virtues of simulation games^13^, a measure of power in game dynamics similar to our definition of gamification badges^14^, a critical overview on the role of optimization models in bench-marking development goals in urban planning^15^, a gamified participatory design/planning framework^16^, and a succinct overview of complexity in urban planning^17^. Correspondingly, the purpose of the proposed game is to provide a non-reductionist model for decision-support in complex decision-making problems concerned with spatial design with constrained resources and a multitude of model sustainability goals (see another game concerned with multi-actor sustainable development in Monechi et al.^18^). The specific sustainable development goals are defined as instances of three archetypal categories, for each of which we have considered a model example:

- social-economic equity: w.r.t. fairly distributing costs and benefits of a development. This objective is ensured already by proposing a participatory opinion pooling mechanism (the term opinion pool dates back to Stone^19^ and De Groot^20^);
- economic-environmental efficiency: w.r.t. scoring the change of allocation per site, and the degree to which the massing distribution blocks the solar potential of the neighbourhood;
- environmental-social comfort: w.r.t. stated preferences (closeness ratings) between the compartments of the district as well as daylight potential of the district.

The proposed game was conceived as a modular and scalable platform that could incorporate various types of evaluation mechanisms on a Digital Twin of an urban district for prototyping Spatial Decision-Support Systems.

A key factor in forming the proposed gamified ’social choice mechanism’ (q.v. Jackson’s definition^21^ & a similar recent formulation^22^) is the subtle difference between optimisation and gamification (regulated group decision-making with scoring mechanisms) approaches to such policy, planning, and design problems involving resource allocation. As stated by Bots and Herman^7^, if stakeholders know each other’s controls and interests and if they agree on fixing some average of these interests and weights of criteria, then the negotiation might be modelled as a puzzle for which some optimal solution can be found. But if there are uncertainties in the definition of the problem and different views towards the objectives, especially if there are power differences, then stakeholders (hereinafter referred to interchangeably as agents or actors) will play strategic ‘games’ that may produce complicated outcomes, better or worse than ideally would be possible depending on whether they would be cooperative or overly competitive. To this end, we propose to measure some game-theoretical indicators of cooperation (contribution to the common objective) and competition (sagacity for achieving one’s ends without much means) to positively reinforce constructive negotiations during the game (vide infra, Fig. 7).

As has been argued by the Nobel Laureate Herbert Alexander Simon^23^, it is common knowledge that one can only refer to ‘the optimal’ in the presence of a single objective. In the so-called multi-objective optimization problems, the reality is that whether we use games, heuristics, meta-heuristics or even mathematical programming methods from Operations Research, we can only ’satisfy’ the problem as formulated in the presence of various simplifications, abstractions, and approximations, but ’the optimal solution’ does not exist as such. Thus, especially if the decision outcome is to be accepted by a group of human actors, playing a purposeful game or going through a structured negotiation process can be arguably more relevant and effective than attempting to reduce a multi-actor multi-criteria decision-making problem into a multi-objective optimization problem.

It is noteworthy that the ideas of utilizing simulation games for understanding decision-making processes and game theoretical approaches to city planning date back at least to the 1970s^13^. Susan Batty clearly describes the kind of complexity arising out of uncertainties concerning the decisions, agendas/value systems, and the costs/benefits of one actor’s decisions for oneself and the influence of the decisions of other actors on one’s interests^24^.

Michael Batty’s formative works on this subject area reveal how the complexities arising out of the spatial context of the problems make the resource allocation problems more challenging and mathematically interesting at the same time^25,26^. In “Evolving a Plan”^27^ Batty proposes a process of Opinion Pooling dating back to French 1956^28^, and Harary 1959^29^, based on his earlier idea of Markovian Design Machines. The typical problem addressed in that book chapter is a recurrent theme in Batty’s work pertaining to the human complexity of multi-actor (multi-agent) decision-making and finding a satisfactory plan of actions (resource allocation) with respect to a set of objects (factors in his formulation). The readers who are interested in the mathematical analysis of opinion dynamics are referred to the work of Jia et al.^30^. The basic set-up of the problem scrutinized by Batty is essentially a problem of resource allocation to multiple objects/sites of interest in an urban redevelopment setting. However, without loss of generality, a similar process can be applied to non-spatial problems of planning accordingly. In fact, what is presented here can be thought of as an extension and a generalization of the work of Batty on Markovian Design Machines and their application to spatial design problems. Our extended problem formulation, in particular, considers that there might be different colours (sorts) of resources to be allocated to a particular target site (could also be a part of a portfolio or any such object of interest).

The main novelty of the proposed framework is a comprehensive mathematical formulation of the spatial design problem at the scale of neighbourhoods that is embedded within a gamified approach to decision-making. This mathematically explicit formulation allows for incorporating methods for multi-criteria ex-ante assessment of abstract spatial designs in a voxelated 3D environment. Additionally, the same formulation is the backbone of methods for fair participation of multiple actors in the decision-making process with a gamified consensus-building mechanism and automatic scoring mechanisms for encouraging proactive participation.

## Proposed framework

Here we present our proposed framework for structuring such generic problems in participatory spatial configuration problems.

### Problem statement

**Figure 1:**
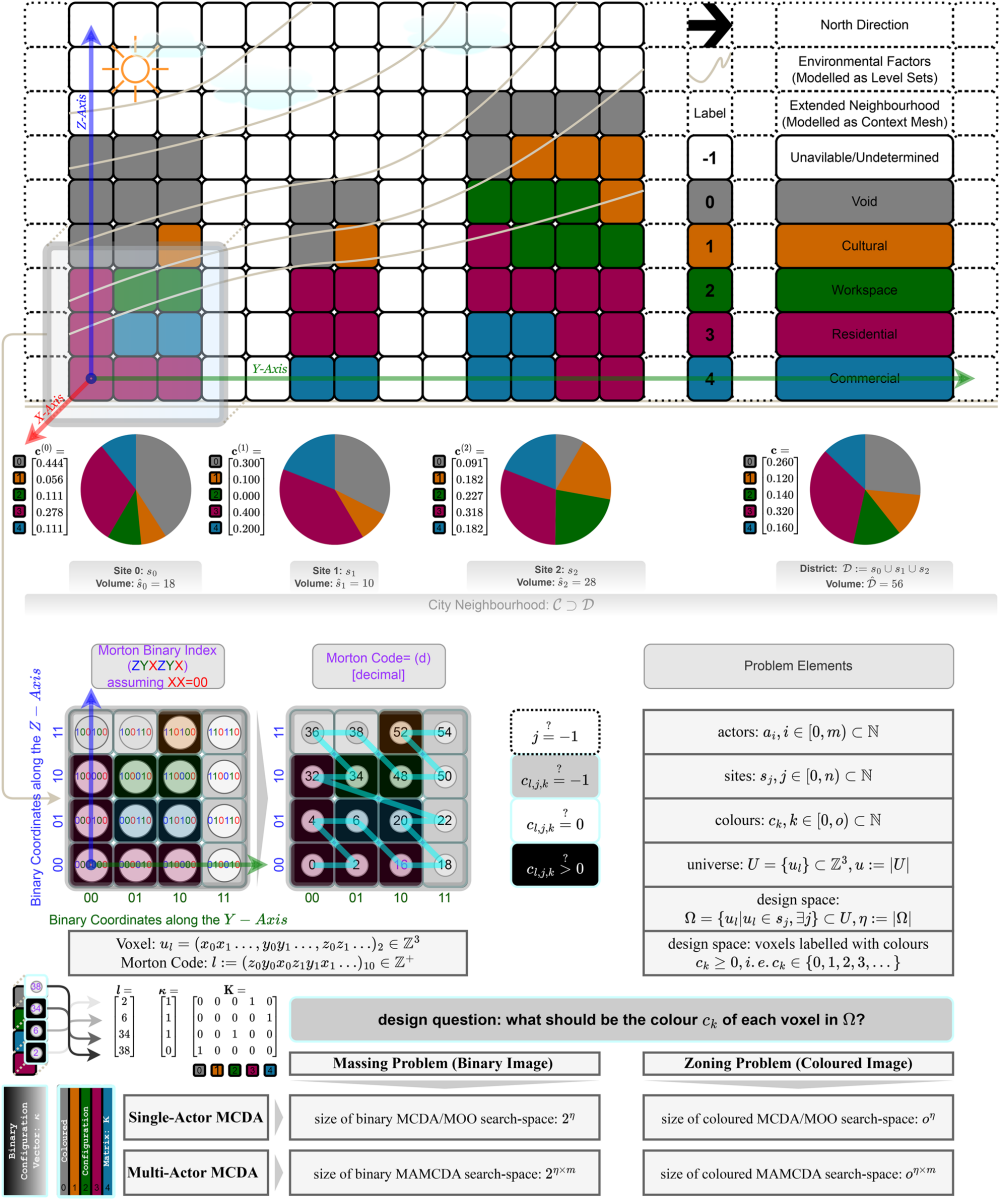
Illustrated formulation of a toy-problem.

The game is to facilitate participation in decision-making for three types of design/planning problems dubbed as pre-planning, planning, and massing problems. Note that the generic design problems dubbed here as massing and zoning are also known by other names such as configuration problems (q.v. an influential framework by Yona Friedman^31^ and a congruent definition in a generative design framework^32^). Although it is important to state the preconditions and assumptions underlying the problem definitions, in the interest of generality, the paper directly goes into the most abstract definition of each problem, for a visual summary see Fig. 1 and see the section Problem-Specific Settings pursuant to the three identified problems.

#### (A) The pre-planning problem

This problem concerns the collation of stated preferences from a group of actors, with the objective of building a consensus by simulating a negotiation and averaging process through a Markov-Chain (Opinion Pooling). The problem is defined as determining the amount of ’investment’ in each category of investment objects (hereinafter referred to as colours), which in this case refer to the distinct types of spaces designated for accommodating different activities (e.g. residential, commercial, cultural, or other sorts of spaces, this is known as a Programme of Requirements in the design and planning jargon). Given a set of such colours, and a set of portfolios (sites or buildings in this case), the actors are to decide how much of each type of investment must be made in each portfolio (how much of each type of space in each site/building). What distinguishes this from a trivial problem of averaging the votes is that the actors have various degrees of interest and control over these various types of investments, denoted respectively by the three-dimensional matrices $\textbf{X}_{m\times n \times o}:=[X_{i,j,k}]_{m\times n \times o}$ and $\textbf{C}_{n\times m \times o}:=[C_{j,i,k}]_{n\times m \times o}$, where *m*, *n*, and *o* respectively denote the number of actors, sites, and colours. The solution to this problem is supposed to be a plan consisting of the amount of desired lettable/saleable net floor space of each colour type per each site, practically a matrix $\textbf{A}_{n \times o}^{(t)}:=[A_{j,k}]_{n \times o}^{(t)}$, where *n* denotes the number sites and *o* denotes the number of colours, and the superscript (*t*) denotes a time stamp referring to the discrete time of the game, colloquially referred to as a round of playing.

#### (B) The planning problem

The planning problem concerns finding the exact amount of volume of each type/colour of space in each site, as closely as possible to the decided amount of net (lettable) coloured areas, matching the prescribed totals for each colour on the entire site, and scaled aptly to integer volumetric quanta in order to yield the expected amounts of net lettable floor area per colour (apropos the consensual decisions of the actors). Additionally, a given set of scaling factors indicates the volume required per each type of coloured space for realizing the desired net floor areas per site, and so, from this point forward the distributions concern the volumetric spaces rather than surface areas.

Whilst the exact values of the latter scale factors are irrelevant to the subject matter of the paper, their practical existence and difference in terms of gross volume to net lettable areas is undeniable and thus typical values are considered to ensure the generality of the formulation. These scale factors, however, are treated as the “advanced settings” of the game and set by the game master rather than the participant players. The adjustment of the local mixes of colours to the expected global mix ratios, dubbed as the District Level Program, denoting Required Surface-Area per Color $\textbf{y}:= [y_{k}]_{o \times 1}$, is performed by means of the Iterative Proportional Fitting. Two additional variable bounds are to be respected in this problem, namely the maximum building height and maximum gross floor area per site. The solution to this problem is a plan indicating the integer number of volumetric cells of space to be built of each colour in each site; this is formally a matrix $\textbf{V}_{n \times o}^{(t)}:=[V_{j,k}]_{n \times o}^{(t)}$.

#### (C) The massing problem

Given the total quanta of the volumetric coloured spatial units per site, the polygonal surface geometry of each site, a regular grid of volumetric spatial units (volumetric pixels or voxels), stated weights of importance of a number of massing quality criteria, procedures for ex-ante assessment of the said quality criteria, a ’3D context mesh’ (a discrete surface consisted of vertices and faces commonly known as a 3D city model, denoted as $\mathcal {M}=(V,F)|V\subset \mathbb {R}^3$) as to which some costs are to be computed, the problem is to determine the shape of a voxelated volume, i.e. the index of the coloured voxels, per each voxelated site so as to satisfy the quality criteria as optimally as possible. Even though the coloured version of this problem (hereinafter referred to as zoning) goes far beyond the scope of this paper, in the latest implementations of the game we have added a simplistic zoning procedure that determines the exact location of each colour within each site for better illustration of the outcome. However, the exact placement of colours within each site, as long as the total amount of coloured space has the same outer envelope shape, does not affect the aggregate quality criteria reported as objectives ($\textbf{q}$, vide infra (C) MAGMA: multi-attribute gradient-driven mass aggregation), and so, the massing problem is one level of abstraction higher than the zoning problem. The solution to the massing problem is in the form of the Mutually Exclusive and Collectively Exhaustive (MECE) indices of coloured voxels in the discrete image of the district, considering a dummy-undefined colour for the uncoloured (undesignated) spaces (see Fig. 1).

#### Problem-specific settings

An urban district with a mix of already existing uses is to be densified to accommodate a larger group of incoming inhabitants. A group of actors is to decide on the colours (i.e. the programmatic allocation of labels) of a number of sites comprising a larger district. Each colour has a different lettable/useable area to volume ratio, due to average space height, different needs for corridor space, and alike.The ratio of Lettable Floor Space (LFS) to Gross Floor Area (GFA) is different and given per colour (we regard such adjustments as advanced settings). The cost of change (implied retrofitting, demolition, or otherwise) is also different and assumed per site and color (another set of items from advanced settings). Additionally, each site has potentially different design codes that can be translated to maximum allowed height or a buildable volumetric envelope. The actors have various levels of control and interest (varied per color) over the district, specified per parcel of land.

The actors can specify their intent (proposal) for the ideal mix of uses (colours) to be allocated to each site. They can specify the desired levels using levels ranging between 0 and 1, which are relativised as portions of colours or coloured surface areas. Similarly, the control of multiple actors over each site has to add up to 100%. Formally, these two conditions translate to the Interest and Control matrices being row-stochastic, which are ensured in the back-end of the system.

The 3D design space is discretised into a regular grid of volumetric pixels (voxels) indexed with globally unique addresses obtained as their Morton Codes (see Fig. 1).

### Problem formulation

In the following we shall compare two alternative formulations of the problem and show how the gamified negotiation problem differs from the optimization problem formulation (cf. Illustrations in the supplementary materials and the Fig. 1 for complexity analysis w.r.t. problem size in MOO and MCDM settings).

#### As an optimisation problem

Given a set of locations with known areas, a regularly discretized 3D spatial domain, a programme of requirements for the district consisting of the said locations specifying the amount of surface area per programmatic label (colour) and the maximum amount of built area per site, it is desired to find the most satisfactory programmatic allocation for the entire district subject to the following constraints and optimality criteria (if weighted similarly by all actors involved):

- Constraints
  - The total allocated area per color must be the same as the given district-level programme
  - The maximum allocated area per site must not exceed the maximum allowed Floor Space Index per site
- Objectives (illustrative)
  - Maximizing the visibility of the sun, the sky, a landscape object of choice (for the district and its neighbourhood within a radius) (figuratively introduced as goals related to Planet & People).
  - Maximizing the similarity of the closeness rates after allocation with the initial given closeness ratings in a REL chart (figuratively introduced as goals related to People & Prosperity).
  - Minimizing change of area allocation per site (figuratively introduced as goals related to Prosperity & Planet).

#### As a gamified negotiation problem

Even though the single-actor optimization problem is not directly addressed in the paper, it is necessary to consider it as a baseline for understanding the size of the problem. It is straightforward to see that the size of the search space, i.e. the complexity of finding a solution (a configuration satisfying the constraints and objectives) for the problem by mere chance, e.g. for a ’monkey behind a type-writer’, corresponds to the number of possible configurations in the discretised domain, that is $2^\eta$ for the massing (black & white colouring) problem. However, there are also agendas and different value systems complicating the problem, thus the size of the search space rises to $2^{\eta \times m}$ (for *m* nitpicking participants without the game mechanisms, that is, see Fig. 1).

To positively reinforce constructive negotiation activities two badges are defined to be issued in each round of the game to the most cooperative player and the most competitive contributor based on a definition of Power Surplus (Dearth) that goes beyond the simplistic definition of winners and losers based on closeness of the final decision to the decision of the actor. In the gamified negotiation problem, there exist *m* opinions on the $n\times o$ distribution of colours on sites. After the stages of Opinion Pooling and Iterative Proportional Fitting (explained further), there will be one such distribution of coloured volumes on sites that can be compared to the expressed opinions of each one of the actors to determine the gainer, the player and the contributor of the round as follows (details further explained in the supplementary materials):

The “Gainer of the Round” is the actor with the most similar interest matrix to the collective decision, formally:

1 $$\begin{aligned} \arg \min _{i} \Vert \textbf{X}^{(t)}[i,:,:]-\textbf{A}\Vert _{F}. \end{aligned}$$

The badge of honour “Player of the Round” is defined as the actor with the most similar pattern of ‘the negative parts of their Power Surplus matrix’ (dubbed $\mathbf {\pi }_{\ominus }^{(t)}$) to the collective decision, or formally as:

2 $$\begin{aligned} \arg \min _{i} \left[ \pi _{\ominus }^{(t)}[i]\right] _{m\times 1} \end{aligned}$$

The badge of honour “Contributor of the Round” is defined as the actor with the most similar pattern of ‘the positive parts of their Power Surplus matrix’ (dubbed $\mathbf {\pi }_{\oplus }^{(t)}$) to the collective decision, or formally as:

3 $$\begin{aligned} \arg \min _{i} \left[ \pi _{\oplus }^{(t)}[i]\right] _{m\times 1} \end{aligned}$$

**Figure 2:**
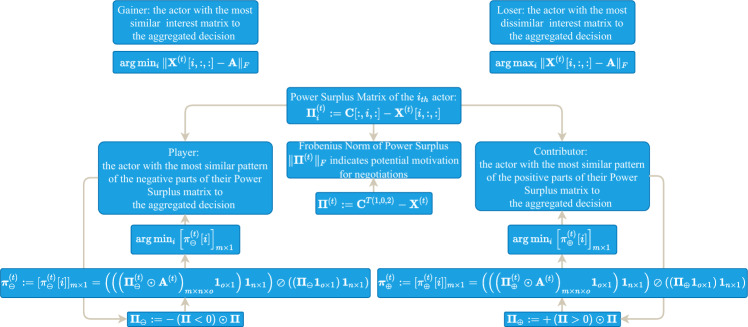
Flowchart indicating the derivation of the Gainer (top-left), Player, and Contributor badges of the game. The Loser badge is not communicated.

While the derivation of the gainer/looser badges is trivial, the player/contributor badges have been derived through a complex process illustrated in Fig. 2. See the Supplementary Information document for the derivation process.

**Figure 3:**
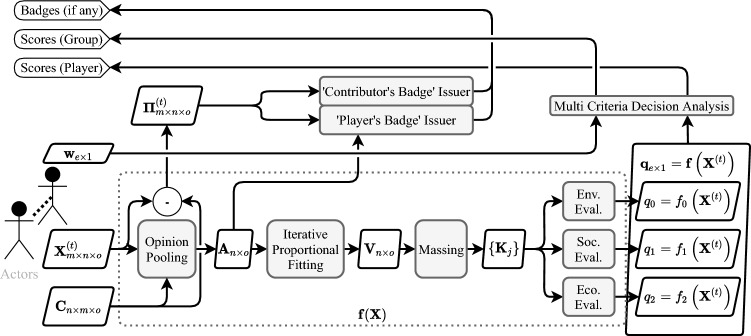
Illustrated formulation of the problem as a gamified negotiation problem, cf. a more detailed schema and an alternative formulation as a multi-objective optimization in the Supplementary Information.

In this formulation, we propose to first build a fair consensus between the actors and motivate them to engage in negotiations by issuing the badges introduced above and informing them in the meantime on the environmental, economic, and social quality of the allocation decision emerging out of the expressed opinions (weighted individual and group scores). The game engine features the following generative process in each round of voting (see the schematic data-flow diagram in the supplementary materials for a visual overview).

## Proposed methods

Here we introduce the proposed processes for participatory design of a city neighbourhood, from the basic discretization of design for converting it into a Multi-Criteria Decision-Making (MCDM) problem, to the creation of a consensus-building procedure (Opinion Pooling), the adjustment of the portfolio according to a [hypothetically] prescribed master-plan of the area by a municipal authority (Iterative Proportional Fitting), generative massing based on a Fuzzy Multi-Criteria Decision Analysis, a very basic (illustrative) zoning process formulated as a clustering problem, and the gamification of the process by encouraging strategic thinking through the issuance of mathematically defined badges dubbed: the gainer, the contributor, and the player of the round.

### Gamified generative design

The following processes are proposed to solve the three problems identified in the framework: (A) Pre-Planning, (B) Planning, and (C) Massing. These processes are generative in that together they generate more information content than they receive from the players and they are looped through a gamified cycle illustrated in Fig. 3 hence the term gamified generative design.

#### (A) Algebraic opinion pooling

The purpose of this process is to simulate the convergence of a fair/equitable pooling of opinions of multiple actors on the distribution of coloured resources over sites, and produce the matrix $\textbf{A}^{(t)}$. Suppose a group of actors is to decide on some degree of action (an amount of investment) on a group of [portfolio] objects (sites in our terminology). Batty^27^ states that if the actors have all the same degrees of interest and control over the objects/factors in question, then the problem is trivial and simple. However, if the degrees of interest and control are not the same then the problem can be formulated as finding a unanimously agreeable or fair consensus amongst the agents with respect to the amount of change so as to deviate minimally from all expressed opinions based on perceptions of actors on their stakes. In other words, an actor might have a high degree of interest in a factor but little control over it and vice versa. These differences between interests and control levels define the non-trivial bipartite relative interaction networks:

- interactions between actors (agents) over sites (factors); this is dubbed as ‘the primal problem’
- interactions between sites (factors) over actors (agents); this is dubbed as ‘the dual problem’

The different types of interests/investments are emblematically referred to as colours in our formulation. Effectively, we generalize the process by iteratively solving the opinion-pooling problem for each colour. Table 1 summarises the derivation of the generalized algebraic opinion-pooling method, and Algorithm 1 shows it in a reproducible and scalable form.

**Table 1:** A summary of the opinion pooling process.

Problem type	Primal problem	Dual problem
Description	Distribute an investment (colour) amongst actors through an interaction network across sites	Distribute an investment (colour) amongst sites through an interaction network across actors
Network	$\textbf{P}=\textbf{X}\textbf{C}$	$\textbf{Q}=\textbf{C}\textbf{X}$
Markov chain	$\varvec{\alpha }^{(t)}={\alpha }^{(t-1)}\textbf{P}$	$\varvec{\beta }^{(t)}={\beta }^{(t-1)}\textbf{Q}$
Steady state (definition)	$\displaystyle \varvec{\alpha }:=\lim _{t \rightarrow \infty }\varvec{\alpha }^{(t)}$ $\varvec{\alpha }\textbf{P}=\varvec{\alpha }$ $\varvec{\alpha }\textbf{1}=1$	$\displaystyle \varvec{\beta }:=\lim _{t \rightarrow \infty }\varvec{\beta }^{(t)}$ $\varvec{\beta }\textbf{Q}=\varvec{\beta }$ $\varvec{\beta }\textbf{1}=1$
Steady State (solution^33^, pp.250–252)	$\displaystyle \varvec{\alpha }\left[ \left( \textbf{I}_{m\times m}-\textbf{P}\right) |\textbf{1}_{m\times 1}\right] =\left[ \textbf{0}_{1\times m}|1 \right]$ $\displaystyle \underbrace{\left[ \left( \textbf{I}_{m\times m}-\textbf{P}\right) |\textbf{1}_{m\times 1}\right] ^T)}_{\textbf{M}} \underbrace{\varvec{\alpha }^T}_{\textbf{x}}=\underbrace{\left[ \textbf{0}_{1\times m}|1 \right] ^T}_{\textbf{a}}$ $\displaystyle \varvec{\alpha }^T=\arg \min _{\textbf{x}}{\Vert \textbf{M} \textbf{x}-\textbf{a}\Vert ^2_2}$	$\displaystyle \varvec{\beta }\left[ \left( \textbf{I}_{n\times n}-\textbf{Q}\right) |\textbf{1}_{n\times 1}\right] =\left[ \textbf{0}_{1\times n}|1 \right]$ $\displaystyle \underbrace{\left[ \left( \textbf{I}_{n\times n}-\textbf{Q}\right) |\textbf{1}_{n\times 1}\right] ^T)}_{\textbf{N}} \underbrace{\varvec{\beta }^T}_{\textbf{y}}=\underbrace{\left[ \textbf{0}_{1\times n}|1 \right] ^T}_{\textbf{b}}$ $\displaystyle \varvec{\beta }^T=\arg \min _{\textbf{y}}{\Vert \textbf{N} \textbf{y}-\textbf{b}\Vert ^2_2}$
Duality	$\varvec{\alpha }=\varvec{\beta }\textbf{C}$	$\varvec{\beta }=\varvec{\alpha }\textbf{X}$

**Algorithm 1:**
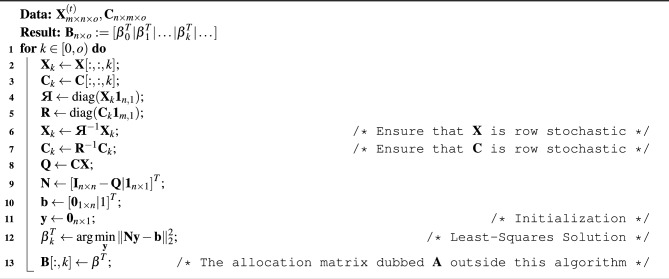
Algebraic opinion pooling (vectorised and generalised to categorical investments).

#### (B) Algebraic iterative proportional fitting

The purpose of this process is to arrive at a matrix $\textbf{V}^{(t)}$ representing the colour volumes for each site with row sums and column sums equalized respectively to the district-level Programme of Requirements and the volume capacities of the sites, while remaining as close as possible to the tensor direction of $\textbf{A}^{(t)}$. A generic problem that occurs in planning with the abstractions presented here is that there might be a prescribed or desired district level programme consisting of a given distribution of the categorical (coloured) investments, while each site (portfolio object) that is being invested in has some capacity for containing coloured amounts of investments, it may locally prefer to have more or less of some amounts. In other words, the players effectively aim for certain local distributions of colours without being able to tediously ensure that the local distributions add up to the two global distributions: a distribution of coloured area amounts for the whole district, and a distribution of the site capacities^34^. This mathematically corresponds to the formation of a “contingency table” or (in the special case that the marginal totals are stochastic themselves) a doubly-stochastic matrix as closely as possible to a (possibly row/column stochastic) desired distribution. The Iterative Proportional Fitting^35–37^ procedure is meant to adjust the entries of the allocation matrix $\textbf{A}=[A_{j,k}]_{n\times n}$, while keeping it similar to the original matrix, such that its row sums and column sums reach the predefined capacity distributions. Algorithm 2 presents our reproducible algebraic method for scalable proportional fitting.

**Algorithm 2:**
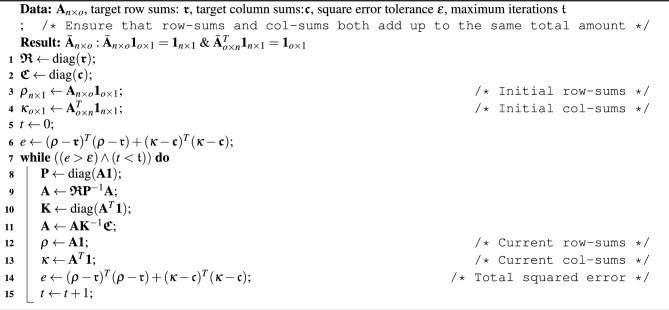
Algebraic iterative proportional fitting.

#### (C) MAGMA: multi-attribute gradient-driven mass aggregation

The purpose of the massing process is to allocate the spaces according to the amounts given in $\textbf{V}^{(t)}$, arrive at a mass-configuration dubbed $\textbf{K}$ (which is only an arbitrarily coloured version of the binary mass-configuration vector $\varvec{\kappa }$) focusing on achieving the highest total (multi-criteria) value for the allocated spaces, w.r.t. the quality-criteria or outcomes of interest given as $\{q_\iota \}$. The method proposed here is based on computing the sensitivities of some aggregate outcomes of interest to the existence or absence of each discrete cellular decision variable (voxel $u_l\in \Omega \subset U\subset \mathbb {Z}^3$) that is indexed with a globally unique index (Morton index). We dub these fields of sensitivities $\varvec{\varphi }^{(\iota )}:=[\varphi _{l}^{(\iota )}]_{\eta \times 1}$.

The major breakthrough of the generative design process is that it utilizes the sensitivities (gradient) of the aggregate evaluation scores (a.k.a. Key Performance Indicators, often abbreviated as KPIs) to decide on the existence/absence of each volumetric cellular space domain (i.e. their density or opacity) at a disaggregated spatial level. Thus, for each volumetric cell we aim to know the contribution of the cell in question to all outcomes of interest. In other words, these two sorts of spatially aggregate and spatially disaggregated evaluation scores are mathematical duals in that the former is the integral of the latter and the latter is (can be considered to be) the gradient of the former.

Thus the following equation shows that the aggregate (integral) outcomes of interest ($\textbf{q}:=[q_{\iota }]_{e\times 1}$) are obtained simply by integrating the gradients (sensitivities):

4 $$\begin{aligned} \varvec{\varPhi }:=[\varphi _{l}^{(\iota )}]_{\eta \times e}, \end{aligned}$$

meaning that if the desegregated sensitivities are known the aggregate KPI can be easily derived as discrete integrals in the form of:

5 $$\begin{aligned} \textbf{q}={1}_{\eta \times 1}^{T}\varvec{\varphi }\mathbf . \end{aligned}$$

The reverse process, i.e. the derivation of the disaggregated evaluation scores however, is often much more complex and domain-specific and thus out of the scope of this paper.

The massing process then forms a multi-criteria total value ($\upsilon _l$) for each cell based on a fuzzy paraboloid AND aggregation (a T-norm function) introduced in^33^, 202 of the disaggregated quality-criteria pertaining to the location in question:

6 $$\begin{aligned} \upsilon _{l}:=\bigcap _{\iota }\varphi _{l,\iota }=\prod _{\iota }\varphi _{l,\iota }^{w_\iota }, \end{aligned}$$

where the eventual weights ($w_\iota$) of quality criteria are the averages of weights submitted by all actors ($\textbf{W}:=[w_{\iota ,i}]_{e\times m}$), i.e.

7 $$\begin{aligned} \textbf{w}:=[w_\iota ]=\frac{1}{m}\textbf{W}\textbf{1}_{m\times 1} \end{aligned}$$

Then the massing process picks the top number of total required voxels in a site *j* that is equal to $\textbf{V}^{(t)}[j,:]\textbf{1}_{o\times 1}$ to find the location of the voxels to be coloured. This result is further processed to create colour zones, albeit only for illustrative purposes. In other words, we perform all quality evaluations only at the level of the massing configuration rather than the zoning configuration (respectively dubbed & illustrated as $\varvec{\kappa }$ and $\textbf{K}$ in our nomenclature). Even in the accessibility score evaluation where we need to know the total volume of each colour in each site, we only project the total colour counts on the single 2D location of the site in question.

### Discrete design evaluation

Currently, the game has three evaluation procedures, namely, the total amount of necessary changes of allocation per-site (indicating potentially demolished property volume, possibly of heritage value), annual solar potential,and efficacy of transportation flows in between the coloured spaces. The latter is chosen to be explained out of these three archetypical evaluation procedures due to its generality and novelty. Without loss of generality, suppose the district provides horizontal access on the ground through some geodesic paths in between the sites and that all coloured spaces are assumed to be projected to a point of entry on the ground as to which their distances are measured (this simplification is necessary for the massing problem at hand and unnecessary for a zoning problem), what is the efficacy of the allocation of colours with respect to the stated preferences for closeness ratings if they are assumed to be estimated transportation flows and the distances considered as transportation costs (as in a transportation problem)?

**Figure 4:**
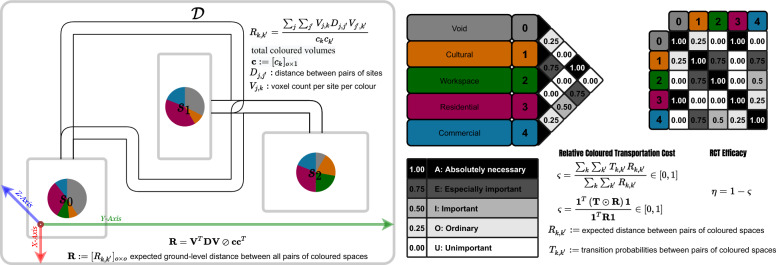
Illustrated coloured accessibility evaluation procedure.

The answer to this question is formulated in two steps illustrated in Fig. 4: Firstly, the relative (expected) distance between the pairs of colours is computed and relativised as to the total amount of coloured spaces present in the allocation–distribution. Secondly, the transportation cost function is formed for the matrix of stated closeness-ratings as a proxy for expected transportation flow-rates between spaces of different colours and the relativised distances between colours are inserted into the equation and the total sum of transportation costs are relativised with respect to the total sum of relativised distances to produce a dimension-less and relative quantity that can be referred to as coloured transportation efficacy, in a manner of speaking. Formally, the first part of the procedure is formulated as:

8 $$\begin{aligned} R_{k,k'}= \frac{\sum _{j} \sum _{j'}V_{j,k}D_{j,j'}V_{j',k'} }{c_{k}c_{k'} }, \end{aligned}$$

where $\textbf{D}:=[D_{j,j'}]_{n \times n}$ denotes the distance between pairs of sites, $\textbf{V}:=[V_{j,k}]_{n \times o}$ denotes the voxel count per site per colour, and $\textbf{c} := [c_{k}]_{o \times 1}$ denotes the vector of total colour volumes in the district. This is summarised algebraically as:

9 $$\begin{aligned} \textbf{R}=\textbf{V}^T \textbf{D}\textbf{V}\oslash \textbf{c}\textbf{c}^T, \end{aligned}$$

where $\textbf{R}:=[R_{k,k'}]_{o \times o}$ denotes the expected ground-level distance between all pairs of coloured spaces. Using the relative distances between coloured spaces computed above, we can form a relative transportation cost dubbed $\varsigma \in [0,1]$ to be minimized as to which the efficacy $\eta =1-\varsigma$. Formally, similar to the cost function of a Transportation Problem in Operations Research^38^, considering the transportation flow-rates $\textbf{T}:=[T_{k,k}]_{o \times o}$ (stated closeness preferences), we form a cost function relativised by the relative distances:

10 $$\begin{aligned} \varsigma =\frac{\sum _{k}\sum _{k'} T_{k,k'}R_{k,k'}}{\sum _{k}\sum _{k'} R_{k,k'}}\in [0,1], \end{aligned}$$

which can be algebraically summarised as:

11 $$\begin{aligned} \varsigma =\frac{\textbf{1}^T \left( \textbf{T}\odot \textbf{R}\right) \textbf{1}}{ \textbf{1}^T\textbf{R}\textbf{1}}\in [0,1]. \end{aligned}$$

## Implementation & demonstrative results

**Figure 5:**
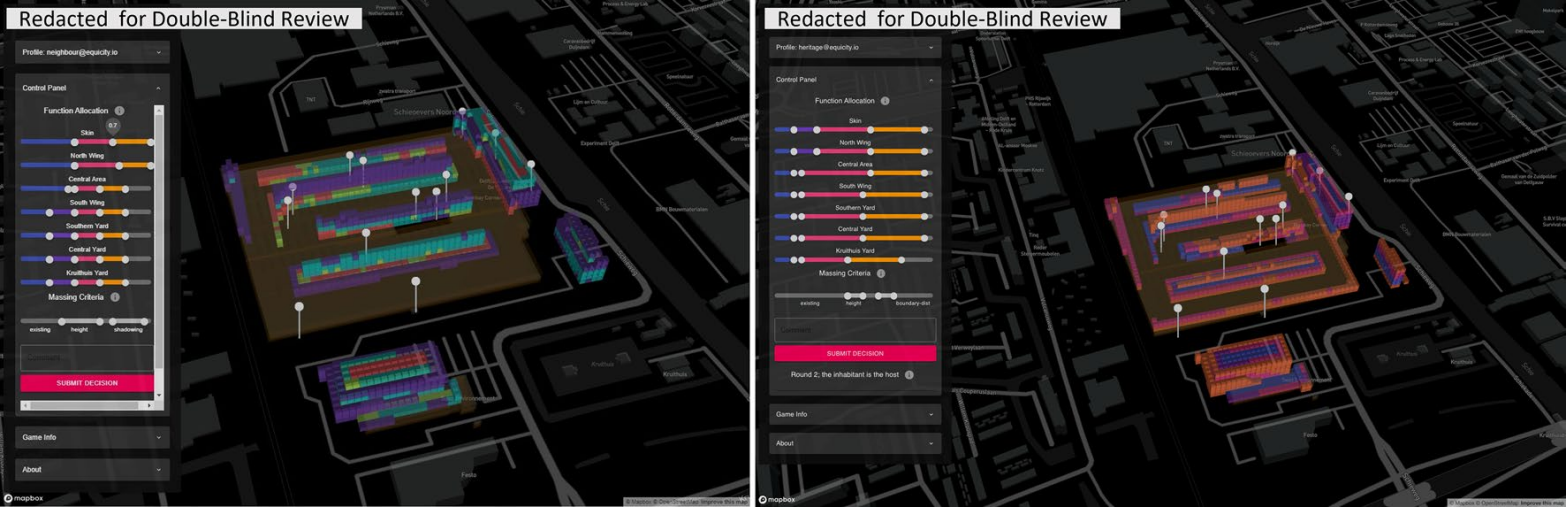
Screenshots of two iterations of game-play, the redevelopment of a former factory into an urban district.

The novel output of our research is the proposed mathematically explicit gamification framework for participatory spatial decision-making. The existing research at the intersection of gamification and decision analysis mainly focuses on increasing the accessibility of the analysis for the players. An example of such an approach is the work of Sousa where he proposes a boardgame setup for the gamification that marries rational systemic planning processes with collaborative processes^3^. Another approach is adding participatory in parallel to the conventional decision analysis as Keseru et. al. do by introducing visioning and scenario building prior to MCDA^39^. In both cases, the researchers are successful in creating an accessible medium of communication for the decision analysis results. However, the lack of a mathematically explicit framework hinders the application of the gamified method to other contexts and application areas. Moreover, the explicit formulation of the problem in the EquiCity framework allows for an objective assessment of the appropriateness of decisions. Lastly, the EquiCity framework and its online web-based implantation allow for scaling the game to larger groups of players and consequently a better representation of the stakeholders (Fig. 5).

The game has been prototyped on a self-developed web-based digital twinning workbench using open-source libraries, tools/services, and freely available platforms: https://equicity.emergentium.io (press Ctrl- upon your visit to adjust the view to your screen). This section gives an overview of the implemented prototyped game and the typical results obtained through multiple test-play workshops with a group of pseudo-actors (role-players) playing an instance of the game set for contemplating the hypothetical redevelopment of a former factory into a dense urban neighbourhood. The participants were encouraged to focus on the challenge of balancing the conservation of the cultural significance (values & attributes) of a plot recognized as industrial heritage through the redevelopment process while satisfying the primary objectives of the development, i.e. adding a significant amount of housing units into the district. The set up the gaming workshop and the data collected from the workshops are explained in detail in the supplementary information file.

The site and the associated fictitious planning problem that were chosen for the development and test cycles are merely to form a vignette for the general idea of participatory generative design of spatial configuration and the challenge of dealing with the idiosyncrasies of the sites particularly in presence of cultural heritage when structuring a systematic participatory design process in the sense of leaving room for negotiations on ad-hoc matters, automating the evaluations that can be automated and providing utmost transparency in the formation and assessment of decisions.

Further, we explain the implementation of the two main components of the game: the interactive interface(front end) and the game engine (back end). The system architecture described here is that of the prototype tested in the final game-play (test) workshop. The workshops required the coordination of a game master who was to oversee the progress made in terms of negotiation dynamics and attainment of sought qualities. Thus, after the final workshop, we developed a game master analytic dashboard containing statistical and data-visualization procedures to observe conclusive reports on the decision-making behavior of the players.

### Interactive interface (front end)

**Figure 6:**
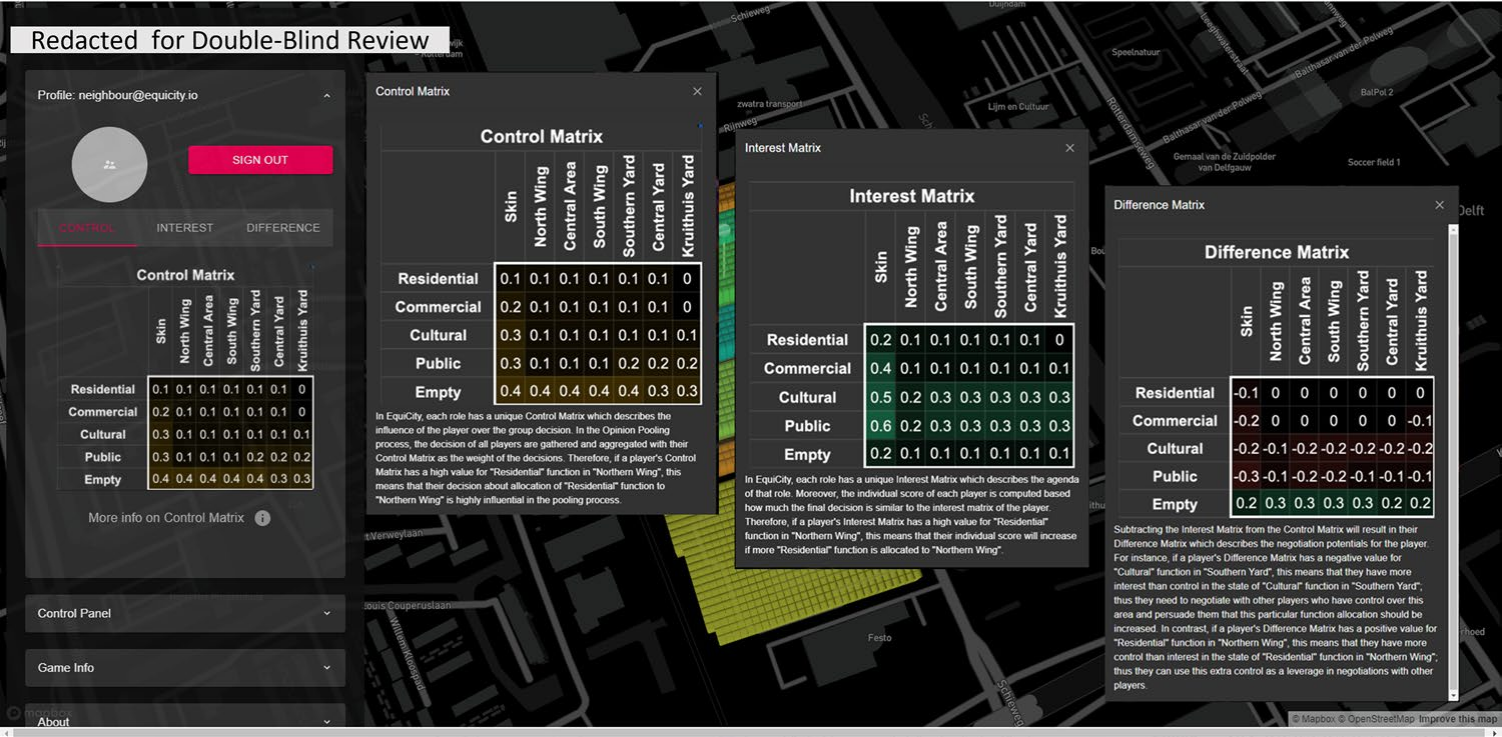
Screenshots of the game interface showing the information panels presented to the players about control, interest, and control–interest difference matrices.

**Figure 7:**
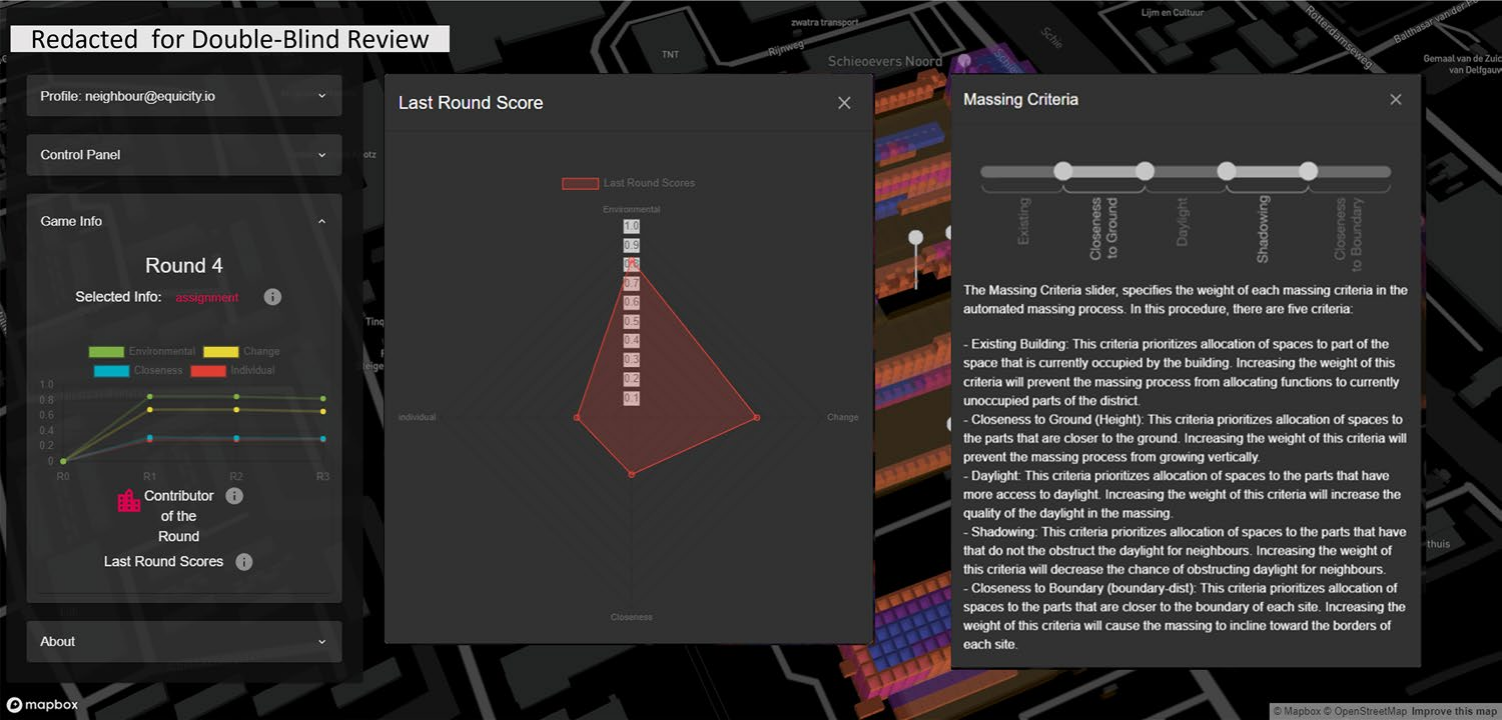
Screenshots of the evaluation output interface of the game and the information provided to players on how to adjust the weights of massing criteria.

The interactive interface allows the players to explore spatial information, environmental analyses, their individual scores, badges, and the agenda of their roles (i.e. the interest matrix at time 0, dubbed $\textbf{X}^{(0)}$ that is supposedly the initial agenda or the mindset of the entity on behalf of whom they act as agents or proxies), and most importantly, to express their decisions for the next round (interests $\textbf{X}^{(t)}$ and weights $\textbf{W}^{(t)}$ for the massing criteria). The interface is web-based, and so, it does not require any prior installation for the participants. Everyone can visit the website to observe the game-play session while it is running and interactively explore the additional information such as spatial analysis and group scores. Each player needs to log-in with their credentials to access their agenda specified as ‘control, interest, and difference matrix (later referred to as the surplus matrix in the text for notational consistency)’ (cf. Fig. 6); make decisions in terms of allocation of colors to sites; and check their individual scores and badges as visible in Fig. 7.

The front end is implemented using React framework in Java-Script; the maps were added using MapBox; geospatial pieces of information were visualized using Vis.gl^40^; and finally scores were visualized using D3^41^.

Within the game-play, in each round, the players input their decisions regarding the allocation of colors to each site through the available sliders in the control panel and specify the weights of massing criteria and add a comment describing the motives behind their decision.(visible in Figs. 6, 6 and 7). Once satisfied with the negotiations, the players submit their decisions and await other players to submit their decisions. Once all decisions are submitted, the game engine kick-starts and goes through a cycle of opinion pooling, proportional fitting, massing, and evaluation. When the cycle is finished all the proceedings are updated in the database and the interface shows the results. These updated data include the spatial distribution of the voxel values, the new mass configuration, the previous massing, submitted decisions (interests, weights), updated scores and badges.

Players, at any time during or after the session, can access aggregated information through the profile and game info menu. Additionally, they can visualize various contextual information and explore the district in the integrated 3D environment, e.g. they can access the annotated heritage attributes and values of the site with their corresponding images and texts by hovering over the pins scattered over the site.

### Game engine (back end)

The Game Engine includes all of the data processing functionalities of the system. Once all decisions are submitted the engine is triggered to perform the following:

First the individual decision of the players ($\textbf{X}^{(t)}_{m \times n \times o}$) are gathered from the database; the opinion pooling is performed to produce the collective decision $\textbf{A}^{(t)}_{n \times o}$; the iterative proportional fitting is performed to produce the Volume-Decision matrix $\textbf{V}^{(t)}_{n \times o}$; the massing is performed to achieve the volumetric configuration of each site $\mathbf {\kappa }$; then the evaluation of spatial massing quality criteria is performed to produce $\textbf{q}_{e \times 1}$; the procedure for allocating game badges is executed; and finally all of the information including the decision variables, evaluation fields $\varphi$, aggregated scores and game-play badges. The disaggregated environmental evaluations are computed per voxel $\varphi _l$ a priori. The evaluation at the aggregate level to produce the vector of multi-criteria outcomes of interest ($\textbf{q}$) is thus only a matter of spatial aggregation (integration). Explaining the exact procedures for computing $\varphi _l$ would go far beyond the scope of this paper and so only one important example of aggregate quality criteria is presented in the paper (coloured accessibility evaluation).

One of the complicated aspects of the game is that the lists of aggregate outcomes of interest and disaggregated fields of quality do not have a one-to-one correspondence, e.g. the aggregate accessibility score is not disaggregated to lead the massing process. In other words, the criteria used for massing are not exactly the same as those displayed as scores. Addressing this issue would fall far outside of the scope of this paper. However, the framework as presented here is to structure such processes and so, without loss of generality, we claim that the proposed examples are generic enough to be representative of the bigger idea of structuring such a complex group decision-making process and nudging it towards sustainable and equitable outcomes.

The game engine is implemented in Python utilizing the following open-source libraries: *NumPy*^42^ for implementing the algebraic processes; *Pandas*^43,44^ for structuring and organizing data; *topoGenesis*^45^ for spatial indexing and topological functionalities of the volumetric units; and finally *HoneyBee*^46,47^ and *EN 17037 Recipes*^48^ for performing visibility and solar analyses.

## Discussion

The paper puts multi-actor consensus and multi-criteria optimization in a challenging spatial context of a design & planning problem. Such problems are notoriously difficult to formulate and tackle, hence the term ’wicked problems’^49^ commonly used to refer to them. In this endeavour, we went beyond such vague notions and addressed the complexity of a generic class of such problems by structuring it mathematically in a novel, straightforward, and open-ended framework without compromising its multiplex sophistication. The algebraic structure of the proposed framework not only makes it elegant and easily explainable; but also very efficient for large-scale implementations that could massively scale up participatory decision-making processes. Without claiming that all the multiple facets of multi-criteria decision-making have been addressed adequately in our experiments, we invite the scientific community to utilize the framework and test the efficacy of various forms of structured group decision-making, especially with a focus on balancing the importance of inter-subjective consensus and optimality w.r.t. objective quality criteria.

With hindsight, following our reflections on the proceedings of the game-play workshops, we were able to identify the contributions and limitations of the proposed serious gaming framework and avenues for further research for generalization of the game to more complex settings or adaptation of the game to other types of spatial or non-spatial Multi-Actor Multi-Criteria Decision Making problems. Namely, in spite of the aim of the project to explicitly address the notion of equity, we did not quantify it, and yet we can envisage that the Game badges can be developed further to produce equity scores. Considering that equity is eventually about fairly sharing both the costs and the benefits of developments, budgeting and cost-sharing definitely need to be integrated into future developments. The implicit meaning of not having considered costs for proposed developments is that the players are acting as idealistic “do-gooders” without worrying about the costs of what they are proposing to be developed. While addressing this issue may be rather straightforward mathematically, incorporating it in the narrative of the game meaningfully would require much effort to ensure consistency. Another generalization that is needed is the consideration of the insistence of some agents on the initial agendas, similar to the formulation of Friedkin et al.^1^. The game is arguably successful in featuring an accessible and scaleable participatory design mechanism. The proposed social choice mechanism allows for simulating otherwise long processes of consensus building by automatically going through rounds of iterations to converge to a point of equilibrium. This effectively allows the players to use the time of the session more effectively for building different kinds of consensual decisions and reflect on the consequences of their choices in the bigger scheme of the neighbourhood and sustainable development goals.

The results of the particular test-case scenario (which is fictitious) suggest that there are some loose ends in particular with regard to the explainability of evaluations and the generation of zones; however, these processes are merely illustrative of the more general idea of a configurator as a gamified “play & score” mechanism. One point of improvement concerns the consideration of MCDA results in issuing the gamification badges of honour. A player might have been very cooperative or competitive in reaching a consensus that is respectively close to their positive power surplus or negative power surplus but that consensual decision may not have necessarily resulted in good outcomes from a multi-criteria decision analysis point of view. In fact, as expected, one of the most difficult (abstract) aspects of the game for the players is to guess the complex associations between their decisions and their measured outcomes (objective functions). This complexity cannot necessarily be alleviated by explaining the advanced settings of the system or the mathematical process, since it pertains to the domain-specific physics of each matter being addressed by each one of the performance/quality indicators. No single player can be expected to be familiar with all of the concepts behind these specific evaluation modules. In fact, the domain-specific knowledge embedded in each one of these evaluation modules is what can be referred to as engineering design expertise, i.e. the know-how rooted in the associations between the decision-variables related to shape and configuration on the one hand and the quality, performance, or functionality of the outcome on the other hand.

The associations between the configurations and their objectively measured qualities (the so-called Key Performance Indicators) are often notoriously baffling to comprehend for even novice or intermediate designers, let alone lay players of the game. Notwithstanding the cognitive difficulty of making the right decisions, the point of devising and playing such a game is to explicate the problem as a decision-making problem to provide direct control for whomever has a stake in the state of the object being designed, be it a neighbour or a prospective inhabitant of the area. However, further contemplation is needed on the distinction between stakeholders who will have to abide by their own decisions and financially partake in the implementation of the decisions and the actors who might have a stake in the development of the site as neighbours (out of the theoretical system). The point is, that it is easy for people who do not have to bear the costs of supposedly good decisions to vote for the most progressive options but nudging the financial stakeholders towards sustainable choices in a persuasive manner is quite a different challenge altogether.

The proposed mathematical framework of EquiCity opens up new research paths in evidence-based and consensual spatial decision-making. Specifically, we can envisage the potential in the following avenues. Firstly, the modularity of the framework allows for the extension and elaboration of the proposed evaluation mechanisms to include more decision criteria. For example, the mathematical explicitness of spatial indexing allows us to incorporate spatial interaction models in the evaluation step to allow for the economic assessment of scenarios. Secondly, the massing process can be made more intelligent to propose zones e.g. by integrating a multi-agent system. Thirdly, we can explore the potential of this framework for a larger and more diverse pool of stakeholders in the decision-making process. Lastly, to make the hard choices for a sustainable future more equitable, we aim to extend this framework by bringing the costs of the choices to the attention of the decision-makers to ensure fairness in decision-making in terms of the proportionality of the costs and benefits for all stakeholders.

### Supplementary Information

Supplementary Information.

## Data Availability

The datasets generated and/or analysed during the current study are available in the EquiCityData repository https://github.com/shervinazadi/EquiCity_Data.
