# Supplementary material for: EquiCity game: a mathematical serious game for participatory design of spatial configurations
Source: Sci Rep. 2024 May 13;14:10912. doi: 10.1038/s41598-024-61093-4 (PMC11091166; doi:10.1038/s41598-024-61093-4)
Supplement: Supplementary file 1 — Supplementary Information. [file 41598_2024_61093_MOESM1_ESM.pdf]

Supplementary Information For EquiCity Game: A Mathematical Serious Game For Participatory Design Of Spatial Configurations

Pirouz Nourian[1,*], Shervin Azadi[2], Nan Bai[3], Bruno de Andrade[4], Nour Abu Zaid[5], Samaneh Rezvani[6], and Ana Pereira Roders[3]

[1]University of Twente, Department of Planning and Geoinformation Management, Enschede, 7522 NH, Netherlands
[2]Eindhoven University of Technology, Department of Built Environment, Eindhoven, 5612 AZ, Netherlands
[3]Delft University of Technology, Department of Engineering and Technology, Delft, 2628 BL, Netherlands
[4]University Portucalense, Department of Architecture and Multimedia Gallaecia, 4200-072 Porto, Portugal
[5]Goldsmiths University of London, Forensic Architecture Research Group, London, SE14 6NW, United Kingdom
[6]DEMO Consultants B.V., Department of Research & Development, Delft, 2628 XJ, Netherlands
[*]p.nourian@utwente.nl

ABSTRACT

This document contains the supplementary information on notational conventions, mathematical derivation of methods, proposed algorithms, implementation details, the structure and typical results of a statistical dashboard for the game master.

Glossary

Nomenclature

Here we present a summary of the essential notations used in the remainder of paper in table 1. Our notational conventions are mostly revolving around the paradigm of vector-computing (algebraic code using vectors, matrices, and tensors), as to which most element-wise expansions and summation notations are omitted for the sake of brevity. We use lower case Latin and Greek letters for scalars, bold lower-case Latin and Greek letters for vectors, bold Latin, Greek, Fraktur, and Cyrillic letters for matrices and tensors, use the slicing 'colon notation' of Golub and van Loan[1] (consistent with NumPy syntax) for denoting matrix/tensor rows, columns, or pages (e.g. $\mathbf{X}[:,:,k]$ denotes the k_{th} page of the tensor $\mathbf{X}$). Dot products are omitted and Hadammard-Schur (element-wise) products/divisions are denoted respectively with $\odot$ and $\oslash$. Augmenting (concatenating/appending) matrices by vectors/matrices is denoted by $|$, e.g. $[\mathbf{A},\mathbf{b}]$ implies that the vector $\mathbf{b}$ is appended to the right side of the matrix $\mathbf{A}$. Unless otherwise stated, all vectors are assumed to be column-vectors.

- **Actors**: define the local tendencies for coloring sites
- **Sites**: need to accommodate all the allocated colors
- **Colours**: represent the discrete types of uses, each with a given surface area from the district-level programme

Symbol	Data Type/Shape	Description
i	integer index of actors	actors: $i \in [0,m) \subset \mathbb{N}$
j, j'	integer index of sites	sites: $j \in [0,n) \subset \mathbb{N}$
k, k'	integer index of colors	colours: $k \in [0,o) \subset \mathbb{N}$
$\mathbf{X}$	$\mathbf{X} := [X_{i,j,k}]_{m\times n\times o}$, a matrix of floats in range of $[0,1]$	Interest Matrix (actors, sites, and colors), no time-stamps for brevity
$\mathbf{C}$	$\mathbf{C} := [C_{j,i,k}]_{n\times m\times o}$, a matrix of floats in range of $[0,1]$	Control Matrix (sites, actors, and colors), fixed in advanced settings
$\mathbf{A}^{(t)}$	$\mathbf{A}^{(t)} := [A_{j,k}]_{n\times o}$, matrix of floats in $[0,1]$ at time t	Collective Decision Matrix, desired areas for sites (sites and colors)
$\mathbf{V}^{(t)}$	$\mathbf{V}^{(t)} := [V_{j,k}]_{n\times o}$, matrix of positive integers at time t	Volume-Decision Matrix, desired volumes for sites (sites and colors)
$\mathbf{c}$	$\mathbf{c} := [c_k]_{o\times 1}$, vector of positive floats	District Level Program as Required Surface-Area per Color
$\mathbf{T}$	$\mathbf{T} := [T_{k,k'}]_{o\times o}$, matrix of positive floats in $[0,1]$	Activity Relations Chart: closeness ratings between coloured spaces

Supplementary Table 1. A summary of the essential notations used in the paper

Definitions

Here we give a brief explanation about the concepts used in this report:

Area Development vs Gentrification

Sometimes a euphemism for a gentrification process, area development usually refers to the development of less-urban areas (such as former military or industry zones) typically surrounded by newer urban developments into new neighbourhoods, mostly incorporating many sought-after housing units and, if regulations, policies, or activism succeeds, a mix of communal spaces, public spaces, cultural spaces, and commercial spaces. From the point of view of the game an urban area development problem, without loss of generality, is regarded as a spatial investment portfolio-management problem.

Actor vs Stakeholder

An actor is a real/legal person or their proxy that is assumed to be playing the participatory design game. Not everyone who has a stake in the development can be guaranteed to be an actor nor vice-versa. A stake-holder would also get a supposedly fair share of both the costs and the benefits of a certain development. However important this may be for the future generalizations of the game, we have not taken the cost-sharing of the proposed developments into account in the scope of the presented game. Thus the players are referred to as actors, who may or may not have the concern of bearing the costs of developments they propose.

Interest vs Control

An actor may have various levels of financial/personal interest in a certain type of investment (e.g. housing, cultural, commercial, et cetera) but not necessarily the same level of control in terms of power over the final decision over the amount of investment on their matter of interest.

Opinion Pooling

A systematic process of consensus-building (a generalized extension of the process introduced by Batty[2]) in which a point of equilibrium is made iteratively by averaging the votes of the actors for investments on a diverse portfolio of distinct investment objects (figuratively referred to as colours/labels in the game) in a number of sites (could be multiple investment portfolios in a group decision-making on resource allocation as referred to in[3]). This procedure is a Markovian Design Machine as introduced by Batty[4] that functions as a consensus-building procedure (as a basis for a consensus on the voting procedures, referred to as procedural consensus in Friedkin's book on Social Influence[5]).

Iterative Proportional Fitting

In various use-cases such as statistical estimation of population distributions or, as identified in this paper, in planning procedures where there is an aggregate picture of a distribution and one needs to work out a disaggregated tabular picture of a distribution to agree in row-sums and column-sums with the prescribed or expected row-sums and column-sums a procedure dating back to 1940's works of statisticians Stephen[6] & Deming[7], nowadays known as Iterative Proportional Fitting[8] is used to adjust the matrices to ensure that the local disaggregated distribution conforms to the expected/prescribed global aggregate picture.

Generative Massing vs Zoning

Generative Design is an umbrella term that refers to simulation-driven processes of computational design formulated as discrete decision-making/optimization problems (mostly shape or topology optimization) that concern the topology/configuration of a spatial object in terms of a binary mass-void distribution. When a single such distribution/configuration is concerned we can refer to it as a massing problem (*vide infra*). If multiple such distributions are to be concerned simultaneously, one has to deal with a 'coloured' distribution (in a graph-theoretical sense colours are in fact distinct categorical labels represented as integers). The latter type of colourful distribution is referred to as zoning in our jargon. Whilst zoning [categorical] distributions are shown in the paper for illustrative purposes, the qualitative evaluation of a zoning distribution falls out of the scope of the paper.

Performance Evaluation vs Quality Assessment

While the two terms are practically synonymous, the connotation of the former in the literature is related to the so-called high-performance engineering and objective measurement of physical quantities, while the latter is mostly associated with problems involving human factors and uncertain decision-making, thus it is preferred in this context. It must be noted that the kind of assessment performed in the game must be *ex-ante assessment* of potential outcomes based on a priori know, and not *ex-post assessment* of actual outcomes, i.e. a posteriori. Given a representation of the designed urban neighbourhood as a mass-void distribution, typically referred to as an envelope, we can estimate the performance of a hypothetical building complex bound into this envelope e.g. with respect to the solar potential of the building or the efficacy of the spatial distribution of activities with reference to an Activity-Relations-Chart representing preferred closeness ratings between the constituent spaces of the neighbourhood. The presented evaluation criteria are merely there to exemplify a broad variety of other such criteria that could be considered for evaluation mass (black & white) or zone (coloured) configurations.

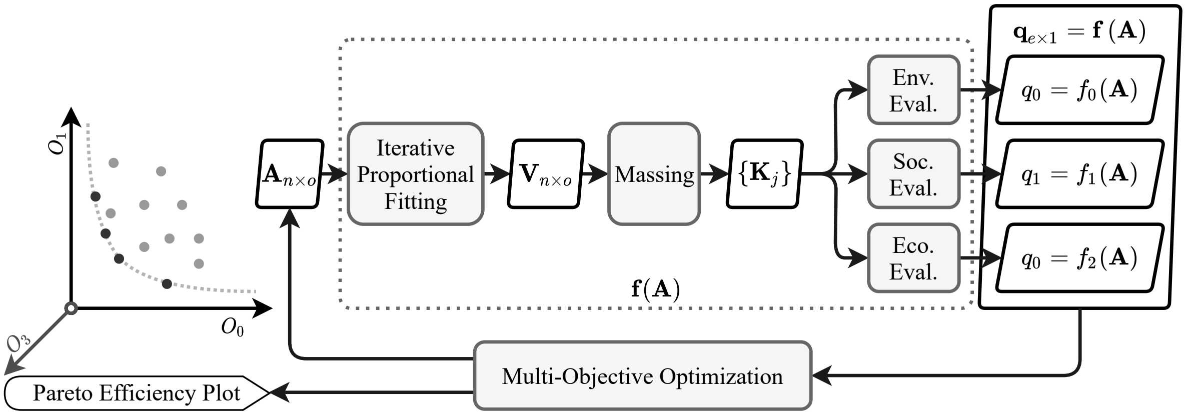

Supplementary Figure 1. Illustrated formulation of the problem as an optimisation problem (not solved in this paper, *vide infra* the gamified negotiation problem)

Optimization Objectives/ Quality Criteria/ Configuration Scors $f_g(\mathbf{A})$:

outcome : $\mathbf{q} := [q_0, \ldots, q_\iota, q_{e-1}]^T = [f_0(\mathbf{A}), f_1(\mathbf{A}), f_2(\mathbf{A}), \ldots]^T$

Constraints $g_r(\mathbf{A})$: a solution (assignment) is assumed to be feasible iff it satisfies all the constraints, i.e.

$$\mathbf{A} \in X \iff g_r(\mathbf{A}) \leq 0, \forall g_r$$

worst possible outcome: $\mathbf{q}^{(nadir)} = \inf_g\{f_g(\mathbf{A})|\forall g, \mathbf{A} \in A\}$

best possible outcome: $\mathbf{q}^{(zenith)} = \sup_g\{f_g(\mathbf{A})|\forall g, \mathbf{A} \in A\ \}$

Optimization Problem Formulation:

$$\bar{\mathbf{X}} = \arg\max_{\mathbf{X}} [f_0(\mathbf{A}), f_1(\mathbf{A}), f_2(\mathbf{A}), \ldots]^T$$
$$s.t.\, \mathbf{A} \in A$$

MAMCDA Problem Formulation:

$$\bar{\mathbf{X}} = \arg\max_{\mathbf{X}} g(\mathbf{X}, \mathbf{W})$$
$$s.t.\, \mathbf{X} \in X$$

Weights of Criteria for a Single Actor: $\mathbf{w}_{e\times 1} := [w_0, w_1, w_2, \ldots]^T$

Weights of Criteria for Multiple Actors: $\mathbf{W} := [W_{\iota,i}]_{e\times m}$

An MCDA equity Function: $e(\mathbf{q}, \mathbf{w})$

An MAMCDA equity Function: $e(\mathbf{q}, \mathbf{W})$

Supplementary Figure 2. Illustrated formulation of the problem as a gamified negotiation problem

Multi-Objective Optimization vs Multi-Criteria Decision Analysis

Since the distinction between the connotations two terms is as wide as the gap between their application areas in physics-dominated engineering and Operations-Research/Management-Science, one cannot easily find a clear-cut distinction between the two in the literature. However, for avoiding any confusion, we find it important to state the subtle difference as addressed by H.A. Simon[9], Jackson[10], and Hwang et al.[11], is that single objective optimization (also known as programming, as in Linear Programming, Integer Programming, and Quadratic Programming in Operations Research) or multi-objective optimization formulation of decision making problems is only possible/meaningful in presence of objectivity and existence of a uniform view on the nature of the objectives and measurements/estimations of attributes as to which the objectives are assessed. When there are multiple actors involved, the uncertainties arising from lack of data, the differences of the value-systems of actors, and the game-dynamics of group decision making complicate the process of finding satisfactory decision-alternatives. Hwang and Lin have explained this briefly, by tangentially referring to the rejection of non-dominated solutions in the sense of Pareto-Optimality criterion: "Moving from a single decision maker to a multiple decision maker setting introduces a great deal of complexity into the analysis. The problem is no longer the selection of the most preferred alternative among the non-dominated solutions according to one individual's (single decision maker's) preference structure. The analysis must be extended to account for the conflicts among different interest groups who have different objectives, goals. criteria. and so on[11]." See a comparative formulation of the posed problem in MOO and MCDM form in Figure 2.

Method

The following figure 3 gives an verview of the proposed methods and the way they constitute the proposed generative design game:

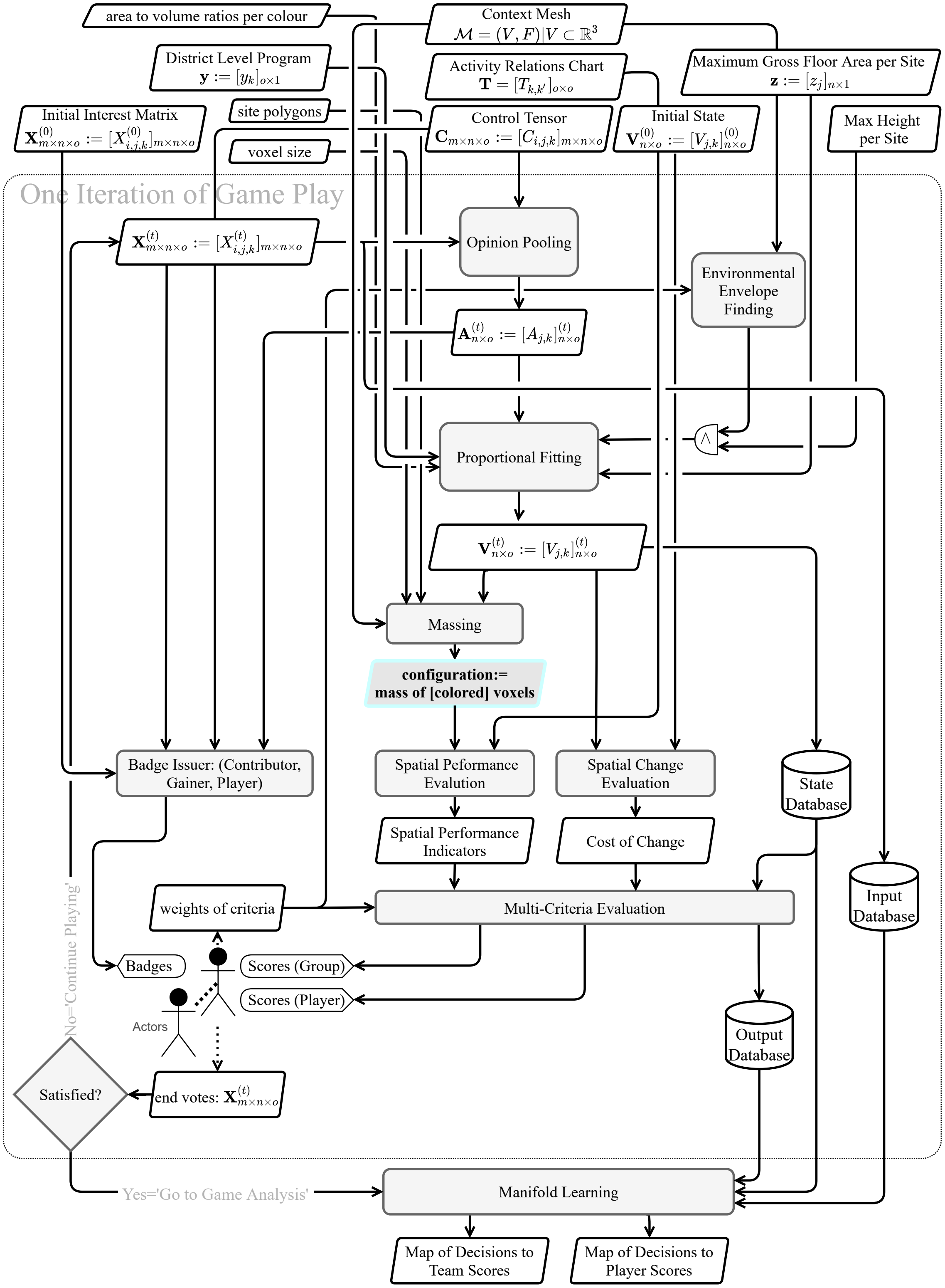

Supplementary Figure 3. Data-Flow of the Proposed Participatory Design Game

Derivation of Gamification Badges

The Winner is the actor with the most similar interest matrix to the aggregated decision, formally:

$$\arg\min_i \|\mathbf{X}^{(t)}[i,:,:] - \mathbf{A}\|_F. \tag{1}$$

The Loser is the actor with the most dissimilar interest matrix to the aggregated decision, formally:

$$\arg\max_i \|\mathbf{X}^{(t)}[i,:,:] - \mathbf{A}\|_F. \tag{2}$$

Out of these two badges, only the first one is communicated by the game engine as the [strategic] gainer of the round. In addition, we propose two more sophisticated badges based on a definition of negotiation power (not dissimilar to the one introduced by Steunenberg et al[12]):

We define a Power Surplus Matrix for the i_{th} actor as:

$$\Pi_i^{(t)} := \mathbf{C}[:,i,:] - \mathbf{X}^{(t)}[i,:,:]. \tag{3}$$

The Frobenius Norm of the tensor made up of the stack of all such matrices $\|\Pi^{(t)}\|_F$ indicates the total motivation for negotiations during the game between those actors with much interest and little control on some investment and those with much control and little interest:

$$\Pi^{(t)} := \mathbf{C}^{T(1,0,2)} - \mathbf{X}^{(t)} \tag{4}$$

The badge of honour "Player of the Round" is defined as the actor with the most similar pattern of the negative parts of their Power Surplus matrix to the aggregated decision, or formally as:

$$\arg\min_i \left[\pi_{\ominus}^{(t)}[i]\right]_{m\times 1} \tag{5}$$

, where

$$\pi_{\ominus}^{(t)} := [\pi_{\ominus}^{(t)}[i]]_{m\times 1} = \left(\left(\left(\Pi_{\ominus}^{(t)} \odot \mathbf{A}^{(t)}\right)_{m\times n\times o} \mathbf{1}_{o\times 1}\right) \mathbf{1}_{n\times 1}\right) \oslash \left(\left(\Pi_{\ominus}\mathbf{1}_{o\times 1}\right)\mathbf{1}_{n\times 1}\right) \tag{6}$$

in which

$$\Pi_{\ominus} := -(\Pi < 0) \odot \Pi. \tag{7}$$

The badge of honour "Contributor of the Round" is defined as the actor with the most similar pattern of the positive parts of their Power Surplus matrix to the aggregated decision, or formally as:

$$\arg\min_i \left[\pi_{\oplus}^{(t)}[i]\right]_{m\times 1} \tag{8}$$

, where

$$\pi_{\oplus}^{(t)} := [\pi_{\oplus}^{(t)}[i]]_{m\times 1} = \left(\left(\left(\Pi_{\oplus}^{(t)} \odot \mathbf{A}^{(t)}\right)_{m\times n\times o} \mathbf{1}_{o\times 1}\right) \mathbf{1}_{n\times 1}\right) \oslash \left(\left(\Pi_{\oplus}\mathbf{1}_{o\times 1}\right)\mathbf{1}_{n\times 1}\right) \tag{9}$$

in which

$$\Pi_{\oplus} := +(\Pi > 0) \odot \Pi. \tag{10}$$

Note that the input of the badge issuer is the matrix $\mathbf{A}^{(t)}$ and not the matrix $\mathbf{V}^{(t)}$. This is because the matrix $\mathbf{A}^{(t)}$ is comparable with $\mathbf{X}^{(t)}$, $\mathbf{X}^{(0)}$, and $\mathbf{C}$ in that all of these matrices contain percentages, but $\mathbf{V}^{(t)}$ contains the actual volumes that ought to be realized.

Derivation of an Algebraic Opinion Pooling Method

Michael Batty, in his seminal paper "Evolving a Plan: Design and Planning with Complexity", proposes a process of Opinion Pooling for urban design based on his earlier idea of Markov Design Machines. The typical problem addressed in this book chapter is a recurrent theme in his works pertaining to the human complexity of multi-actor (multi-agent) decision making and finding a satisfactory plan of actions (resource allocation, i.e. where shall we invest our resources?) with respect to a set of sites or investment portfolio objects (called factors in his formulation).

For convenience, we shall be using Batty's terminology but with a slightly different notation pertaining to our generalized problem.

There exist a set of m actors/agents and n sites/objects. Each actor has:

- a degree of interest $\mathbf{X} = [X_{i,j}]_{m\times n}$, s.t. $\mathbf{X1} = \mathbf{1}$, i.e. this bipartite matrix is row-stochastic, or that the relative interests of each agent over the n objects add up to exactly 100%, and

- a degree of control $\mathbf{C} = [C_{j',i'}]_{n\times m}$, s.t. $\mathbf{C1} = \mathbf{1}$, i.e. this bipartite matrix is row-stochastic, or that the relative controls of the m agents over each object add up to exactly 100%

Respectively the two problems will be solved as below:

- $\mathbf{P} := [P_{i,i'}]_{m\times m} = \mathbf{XC}$: The Markovian interaction probability matrix between agents through their interests and controls over the objects, whose entries represent the relative importance of connections based on the interest & control connections
- $\mathbf{Q} := [Q_{j',j}]_{n\times n} = \mathbf{CX}$: The Markovian interaction probability matrix between factors through the interests and controls of the agents, whose entries represent the relative importance of connections based on the control & interest connections

The Plan Design problem can be defined as 'a process of resolving the differences between agents w.r.t. their planned collective investments on the objects given their different interests and controls'. We can define two processes of "conflict resolution" on the primal or the dual problem.

The Primal Problem: Consider a negotiation process/game in which the agents collectively redistribute an arbitrary/existing allocation of resources according to the expressed interests and agreed controls. *This is opinion pooling across sites over the distribution of 1 colour amongst actors.* Thus, this form of the problem is only theoretically important but not relevant in this case. It turns out that such a process corresponds to an "ergodic Markov chain" that has a steady-state distribution of resources. The arbitrary initial allocation/distribution of resources is dubbed $\alpha^{(0)} = [\alpha_i]_{1\times m}$, i.e. α is a *row vector*. A Markov chain can be formulated as follows:

$$\alpha^{(1)} = \alpha^{(0)}\mathbf{P} \Rightarrow \alpha^{(t)} = \alpha^{(t-1)}\mathbf{P} \Rightarrow \alpha^{(t)} = \alpha^{(0)}\mathbf{P}^t. \tag{11}$$

This Markov process is ergodic under relatively simple conditions and thus it can be expected to converge to a steady-state solution characterizing an equilibrium state in which the relative pooling of the categorical (coloured) resources according to all the agents has stabilized as a function of interaction matrix (that reflects relative interests and controls), i.e. in the steady-state:

$$\lim_{t\to\infty} \alpha^{(t)} = \alpha^{(t)}\mathbf{P}, \tag{12}$$

because, by definition, at the steady state $\alpha^{(t)} = \alpha^{(t-1)}$. Now, if for convenience we denote this steady-state as $\lim_{t\to\infty} \alpha^{(t)} := \alpha$, and so, we can rewrite the last equation as below:

$$\alpha = \alpha\mathbf{P}. \tag{13}$$

In this form, it is easy to see that α is a left-eigenvector of $\mathbf{P}$ or that α^T a right-eigenvector of $\mathbf{P}^T$ with an eigenvalue equal to 1. At the same time, this eigenvector must be a stochastic vector (a discrete probability distribution), i.e. $\alpha\mathbf{1} = 1$. Now, following an algebraic procedure [13, pp.250-252], we can find such a stochastic eigenvector using a standard Gaussian [Least Squares] solver. Consider the steady-state solution:

$$\alpha = \alpha\mathbf{P} \Rightarrow \alpha(\mathbf{I} - \mathbf{P}) = \mathbf{0} \textit{ and } \alpha\mathbf{1} = 1 \tag{14}$$

We can combine these two equations into one system of linear equations:

$$\alpha\left[(\mathbf{I}_{m\times m} - \mathbf{P})\,|\mathbf{1}_{m\times 1}\right] = [\mathbf{0}_{1\times m}|1] \Rightarrow \left[(\mathbf{I}_{m\times m} - \mathbf{P})\,|\mathbf{1}_{m\times 1}\right]^T \alpha^T = [\mathbf{0}_{1\times m}|1]^T. \tag{15}$$

The latter equation can be solved by in the sense of finding a least squares solution using a standard numerical solver for the $\mathbf{Mx} = \mathbf{a}$ (see the complete notation in the Opinion Pooling Table in the paper) to find this stochastic eigenvector, i.e.

$$\alpha^T = \arg\min_{\mathbf{x}} \|\left[(\mathbf{I}_{m\times m} - \mathbf{P})\,|\mathbf{1}_{m\times 1}\right]^T \mathbf{x} - [\mathbf{0}_{1\times m}|1]^T\|_2. \tag{16}$$

The Dual Problem: consider a negotiation process/game in which an arbitrary/existing allocation of values on factors the factors is collectively pooled by agents according to their designated control and expressed interests. *This is opinion pooling across actors over the distribution of 1 colour amongst sites.* Thus, this is exactly the problem we need to solve. It turns out that such a process corresponds to an ergodic Markov chain that has a steady-state distribution of values. The arbitrary initial allocation/distribution of values is dubbed $\beta^{(0)} = [\beta_j]_{1\times n}$, i.e. β is a *row vector*. The rest of the process is similar to the primal problem and so, for the sake of brevity, only the equations are presented: A Markov chain can be formulated as follows:

$$\beta^{(1)} = \beta^{(0)}\mathbf{Q} \Rightarrow \beta^{(t)} = \beta^{(t-1)}\mathbf{Q} \Rightarrow \beta^{(t)} = \beta^{(0)}\mathbf{Q}^t \tag{17a}$$

$$\lim_{t\to\infty} \beta^{(t)} := \beta \Rightarrow \beta = \beta\mathbf{Q} \tag{17b}$$

$$\beta = \beta\mathbf{Q} \Rightarrow \beta(\mathbf{I} - \mathbf{Q}) = \mathbf{0} \textit{ and } \beta\mathbf{1} = 1 \tag{17c}$$

$$\beta\left[(\mathbf{I}_{n\times n} - \mathbf{Q})\,|\mathbf{1}_{n\times 1}\right] = [\mathbf{0}_{1\times n}|1] \Rightarrow \left[(\mathbf{I}_{n\times n} - \mathbf{Q})\,|\mathbf{1}_{n\times 1}\right]^T \beta^T = [\mathbf{0}_{1\times n}|1]^T \tag{17d}$$

$$\beta^T = \arg\min_{\mathbf{y}} \|\left[(\mathbf{I}_{n\times n} - \mathbf{Q})\,|\mathbf{1}_{n\times 1}\right]^T \mathbf{y} - [\mathbf{0}_{1\times n}|1]^T\|_2. \tag{17e}$$

It is straightforward to verify that:

$$\begin{Bmatrix} \beta = \alpha \mathbf{X} \\ \alpha = \beta \mathbf{C} \end{Bmatrix}. \tag{18}$$

From this it is clear that such correspondence not only exists in the two steady states but also during the transient states. In other words, given any distribution of resources over agents or values over the factors, we can find the corresponding dual distribution reciprocally. In particular, given a distribution of resources amongst agents $\alpha^{(t)}$, one can easily find the distributed values of the factors of that iteration time by applying the relative interest of the agents over all factors as captured by the matrix **x**. This is to say that the RHS of the first equation distributes the resources of agents over the factors and produces the values invested in all factors. Analogously, given a distribution of invested values over the factors one can work out how much each agent has invested their resources by using the matrix **Q** (the RHS of the second equation). This also implies that throughout the transient states, the resource and value distributions between two iterations are unequal but get closer to their next iteration values if we feed the next result back into the same pooling process, i.e. make the Markov Chain.

Derivation of an Algebraic Proportional Fitting Method

We have condensed this procedure as an algebraic algorithm as follows: the target row-sums and column-sums are iteratively scaled alternatively with column-relativised (ensured to be column stochastic) and row-relativised (ensured to be row stochastic) versions of the original matrix until the series converges, i.e. the norm of the differences between the actual row/column sums with the target sums becomes negligible. The row-sums and column-sums are denoted as vectors

$$\rho_{n\times 1} := \mathbf{A}_{n\times o}\mathbf{1}_{o\times 1} \;\&\; \kappa_{o\times 1} := \mathbf{A}^T_{o\times n}\mathbf{1}_{n\times 1}, \tag{19}$$

reach target values, denoted as vectors

$$\mathfrak{r}^{(T)} := [\mathfrak{r}_i^{(T)}]_{n\times 1} \;\&\; \mathfrak{c}^{(T)} := [\mathfrak{c}_j^{(T)}]_{o\times 1}. \tag{20}$$

The procedure in vectorized (algebraic) form is to iteratively adjust the row-sums and column sums iteratively until the row sums and the column sums get close enough to the target row sums and column sums. Therefore, in a vectorized form, we can write the row-sum adjustment/fitting operation as a combination of relativisation or conversion to a row/column-stochastic matrix and then scaling the rows from the left or scaling the columns from the right, as explained in Algorithm 2 in the paper.

$[\mathbf{X}[:,:,0]]_{m\times n} =$

```
[[0.25985233 0.09766022 0.64248745]
 [0.04107522 0.44759587 0.51132891]
 [0.1950742  0.0757569  0.72916891]
 [0.44637416 0.4376011  0.11602474]]
```

$[\mathbf{C}[:,:,k]]_{n\times m} =$

```
[[0.27737148 0.12030239 0.15985201 0.44247412]
 [0.26330566 0.20108439 0.2583966  0.27721336]
 [0.13080753 0.03266753 0.49548771 0.34103723]]
```

Primal Problem

e.g. 4 actors,
3 sites,
& 5 colours:
opinion pooling
across sites
over the distribution of
1 colour amongst actors

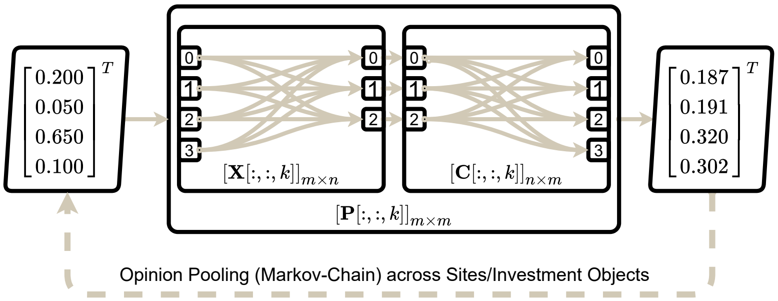

$[\mathbf{P}[:,:,0]]_{m\times m} =$

```
[[0.18183231 0.07188728 0.38511762 0.36116279]
 [0.19613329 0.11164984 0.3755804  0.31663647]
 [0.16943602 0.06252157 0.41205256 0.35598985]
 [0.25421122 0.14548487 0.24191727 0.35838664]]
```

```
The 0_th iteration of pooling results in: array([[0.2 , 0.05, 0.65, 0.1 ]])
The 1_th iteration of pooling results in: array([[0.18172766, 0.07514745, 0.38782844, 0.35529645]])
The 2_th iteration of pooling results in: array([[0.20381533, 0.09739201, 0.34396848, 0.35482418]])
The 3_th iteration of pooling results in: array([[0.20464297, 0.09865253, 0.3426426 , 0.35406191]])
The 4_th iteration of pooling results in: array([[0.20462226, 0.09865897, 0.34270402, 0.35401476]])
The 5_th iteration of pooling results in: array([[0.20461817, 0.09865518, 0.34271236, 0.35401428]])
The 6_th iteration of pooling results in: array([[0.20461798, 0.09865492, 0.34271269, 0.35401441]])
The 7_th iteration of pooling results in: array([[0.20461798, 0.09865491, 0.34271269, 0.35401442]])
The 8_th iteration of pooling results in: array([[0.20461798, 0.09865491, 0.34271268, 0.35401442]])
The 9_th iteration of pooling results in: array([[0.20461798, 0.09865491, 0.34271268, 0.35401442]])
```

Dual Problem

e.g. 4 actors,
3 sites,
& 5 colours:
opinion pooling
across actors
over the distribution of
1 colour amongst sites

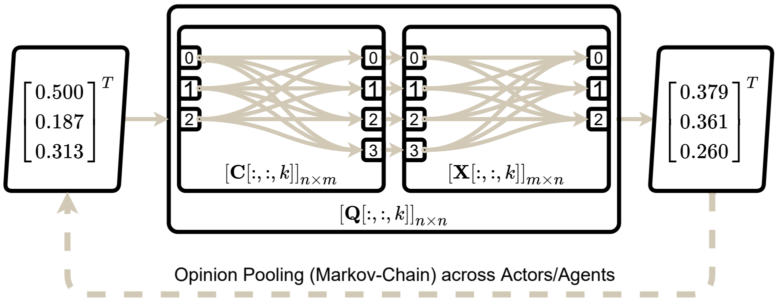

$[\mathbf{Q}[:,:,0]]_{n\times n} =$

```
[[0.30570909 0.28667207 0.40761884]
 [0.25082756 0.25660323 0.49256921]
 [0.28421954 0.21417142 0.50160904]]
```

```
The 0_th iteration of pooling results in: array([[0.5 , 0.1875, 0.3125]])
The 1_th iteration of pooling results in: array([[0.28870332, 0.25837771, 0.45291897]])
The 2_th iteration of pooling results in: array([[0.2817959 , 0.24606603, 0.47213807]])
The 3_th iteration of pooling results in: array([[0.28205858, 0.24504283, 0.47289859]])
The 4_th iteration of pooling results in: array([[0.28209839, 0.24501846, 0.47288315]])
The 5_th iteration of pooling results in: array([[0.28210006, 0.24502031, 0.47287963]])
The 6_th iteration of pooling results in: array([[0.28210003, 0.24502051, 0.47287946]])
The 7_th iteration of pooling results in: array([[0.28210002, 0.24502052, 0.47287946]])
The 8_th iteration of pooling results in: array([[0.28210002, 0.24502052, 0.47287946]])
The 9_th iteration of pooling results in: array([[0.28210002, 0.24502052, 0.47287946]])
```

Supplementary Figure 4. an illustrative example of Iterative Opinion Pooling processes

Game-play

We have put a web-based prototype of the game to test through three participatory serious gaming workshops. The first two workshops were mainly dedicated to testing the prototype and collecting qualitative feedback on the interface and desired features. The last workshop was to obtain structured test data and collect first-hand feedback from role-players on how real stakeholders could/would interact with the system potentially at massive participation scales. For the sake of brevity, in what follows only the set up and the data collected from the last test-play workshop is discussed.

The case study of the workshop was a former sub-urban industrial district in a historical city in the Netherlands. Recently the municipality had decided to redevelop the district with the aim to convert it into a high-density residential urban neighbourhood. This redevelopment process is supposed to add extra *Residential*, *Commercial*, *Cultural*, and *Public* spaces as distinct categories of designated uses (referred to as functions in the architects' jargon) to the district. These four categorical functionalities, in addition to the *Empty*, comprise the five Colors of the game (regarded as categorical data dubbed c_k in the paper). There was already a proposal for the redevelopment of the district by a design firm with an idea to preserve the industrial character of the site as much as possible. Accordingly, we divided the district into seven constituent sites closely following its current spatial composition, with the idea to preserve as much as possible the structure of the district: *Skin*, *North Wing*, *Central Area*, *South Wing*, *Southern Yard*, *Central Yard*, and *Kruithuis Yard*. In the last workshop, five actors played the emblematic roles of *The Mayor*, *The Neighbour*, *The Architect*, *The Inhabitant*, and *The Developer*.

The gaming workshop was held online; each participant joined a shared video call and accessed the web-based interface using their credentials. They had received the credentials together with an invitation and a brochure explaining the interface, the narrative, and the riles of the game, as shown in Figure 5. This setting allowed us to record the proceedings of the workshops not only in terms of interaction data but also the verbal conversations (video link removed for double-blind review).

The session took 150 minutes, including a 40-minute introduction to the game, 40 minutes of playing, a 10-minute break, 40 minutes of playing, and finally, 20-minute feedback and reflection. The participants had no prior knowledge of the game interface.

During this 80 minutes of game-play, the players played three complete rounds. In each round, one of the players was chosen to be the host (negotiation moderator) of the round randomly. The host would start the negotiations with other players to persuade them to vote in favor of his/her interests (represented by their interest matrix). In addition to the interest matrix, players had access to control and power surplus matrices (the latter was called the difference matrix at the time), representing the level of control and mismatch of their interest and control respectively. Based on the difference (power surplus) matrix, players could understand what areas of interest are outside of their control, so they should rely on other players to support them there to steer the design towards their interest matrix; and what areas of their control they have no interest in so they can use as tokens of power barter in their negotiations.

After the negotiations were concluded (if necessary with a time pressure exerted by the game master), the players would proceed to input their decision (effectively update their interest matrices) about the allocation of space in each site to each color, as well as their assigned weights of importance for the massing quality criteria and submit their decisions in the interactive interface. When the system ensures that all players have submitted their decisions in a round, the back-end starts to go through the opinion pooling, iterative proportional fitting, massing, and evaluation processes to provide feedback regarding the aggregate outcome of the collective decisions of players in terms of group scores, individual scores, and individual badges.

The near real-time communication of the outcomes of interest (aggregate quality criteria dubbed as q_ι) marks the end of a round of game-play, after which players can access the scores, badges, and extra info to understand the performance of their decision and contemplate on changing their decisions in the subsequent round accordingly. The game interface provides extra information regarding the spatial quality criteria used for massing (in pop out windows). This type of information, i.e. the association between the individual decisions and the aggregate outcomes of interest for the group was found to be the most abstract for the players to grapple with in all workshops. We presume that this complex association is what is colloquially known as complexity of design in terms of the difficulty of adjusting the decisions for obtaining a satisfactory balance on measurable outcomes of interest.

Game Master Dashboard

To assist the game master in assessing the game play process, we put forth a set of statistical workflows that provide insight into how the players are making decisions, how effective these decisions are, and finally, whether the decisions are improving during the game play workshop. To this end, we restate the following assumptions as hypotheses to be tested statistically:

- Decision Homogeneity: Decisions are not homogeneous across actors, w.r.t. sites, and colors.
- Decision Change: Decisions are changing throughout the rounds.
- Discussion Time: Longer rounds will yield a more significant improvement in the decisions.

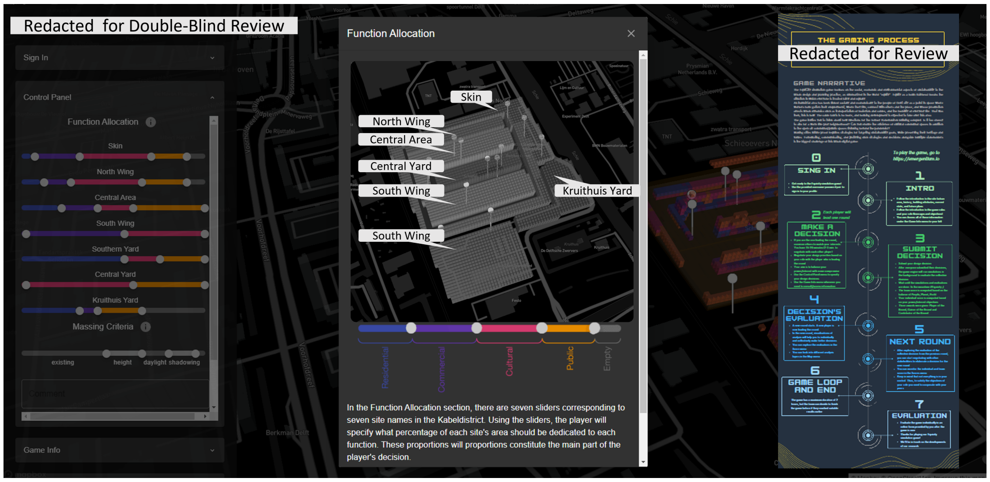

Supplementary Figure 5. Screenshots of the game interface and its infographic brochure

Given that in each round, each actor makes a decision about how much space in each site is allocated to each color, the decisions are formatted as $\mathbf{X}_{\tau\times m\times n\times o}$ where τ is the counter of discrete time iterators or the number of played & recorded game-rounds, m is the number of actors, n is the number of sites, o is the number of colors. Furthermore, since scores are calculated in each round per each criterion (three criteria in addition to m individual criteria showing the attainment of each individual as the extent to which they have been a gainer in the previous round), the scores are formatted as $\mathbf{O}_{t\times(m+3)}$. It must be noted that the framework is devised to be scalable for mass-scale participation and the proposed statistical hypothesis testing procedures would eventually require more data to be conclusive. Nevertheless, the procedures are illustrative of the kind of information and insight that the game master could receive to utilize for activating the negotiations.

Decision Homogeneity

We assume that decisions are not homogeneous across actors, w.r.t. sites, and colors. The importance of this assumption is that if the decisions turn out to be homogeneous, then the multi-actor complexity of the game is trivialized. To test this hypothesis, firstly we check the homogeneity of the data for equal variances using Levene's W.

Parameter	Value
Test statistics (W)	0.726
Degrees of freedom (df)	139
p value	.982

Supplementary Table 2. The result of Levene test for the final workshop game-play data

The data-set with three-way design has equal variance with Levene test ($W = 0.727, df = 139, p = .983$), which fulfills the assumption for ANOVA.

Source	SS	df	MS	F	p	η^2
Actor	0.370	4	0.093	23.630	.000	.252
Site	0.066	6	0.011	2.794	.012	.056
Color	0.665	3	0.222	56.636	.000	.378
Actor * Site	0.286	24	0.012	3.044	.000	.207
Actor * Color	1.274	12	0.106	27.106	.000	.537
Site * Color	0.972	18	0.054	13.793	.000	.470
Actor * Site * Color	1.619	72	0.022	5.742	.000	.596
Residual	1.097	280	0.004	NaN	NaN	NaN

Supplementary Table 3. The result of three-way ANOVA test for the final workshop game-play data assessing the effect interaction of Actor, Site, and Color

The three-way ANOVA shows that the main effects of Actor, Site, and Color are significant: $F_{\mathrm{a}}(4,280) = 23.630$, $p < .001, \eta^2 = .252$; $F_{\mathrm{s}}(6,280) = 2.794$, $p = .012$,$\eta^2 = .056$; $F_{\mathrm{c}}(3,280) = 56.636$, $p < .001, \eta^2 = .378$. The two-way interaction effect between Actor-Site, Actor-Color, Site-Color are all significant: $F_{\mathrm{a*s}}(24,280) = 3.044$, $p < .001, \eta^2 = .207$; $F_{\mathrm{a*c}}(12,280) = 27.106$, $p < .001, \eta^2 = .537$; $F_{\mathrm{s*c}}(18,280) = 13.793$, $p < .001, \eta^2 = .470$. And the three-way interaction effect among Actor, Site, and Color is significant: $F_{\mathrm{a*s*c}}(72,280) = 5.742$, $p < .001, \eta^2 = .596$.

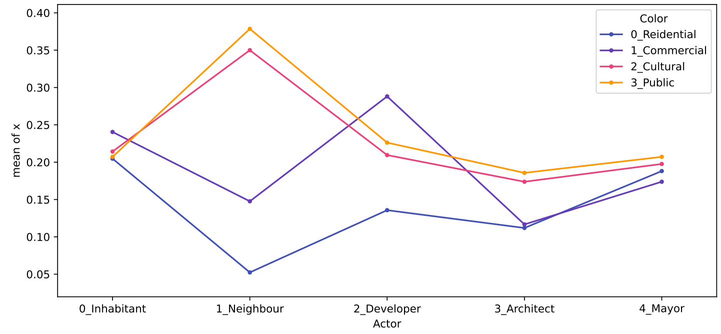

Supplementary Figure 6. Interaction plot of actors and colors, revealing that amongs actors some have aimed for uniform distributions, (the inhabitant and the mayor) and that the neighhbour seems to have had the sharpest distinction of spatial categories in their treatment of colours

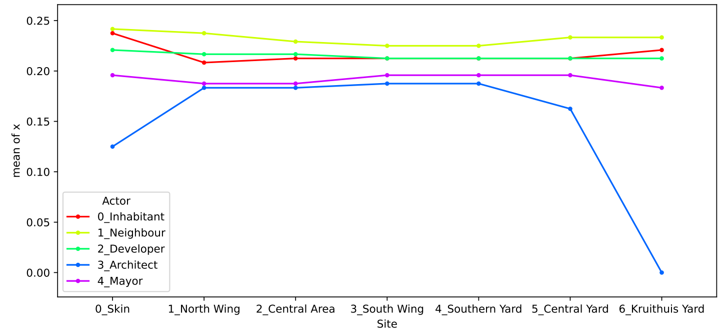

Supplementary Figure 7. Interaction plot of sites and actors, revealing e.g. that the architect has made the most distinct spatial allocation decisions to the sites, apparently in an attempt to achieve a certain shape for the resultant mass configuration

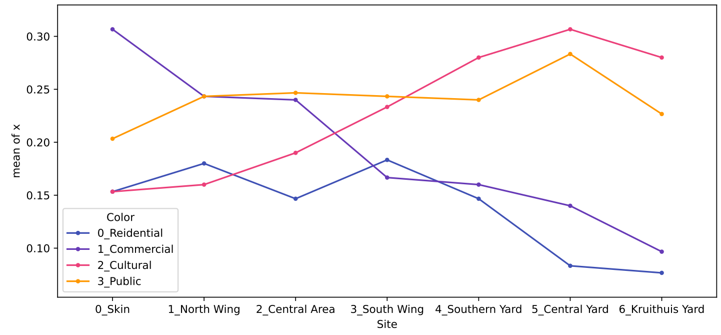

Supplementary Figure 8. Interaction plot of sites and colors, revelating that the sites have been treated distinctly w.r.t. their colour distributions by all actors throughout the played rounds

A post-hoc analysis is conducted using Tukey-HSD test to investigate the inter-relationships of Actors, Sites, and Colors.

A	B	mean(A)	mean(B)	diff	*SE*	*T*	*p*-tukey	hedges
0_Inhabitant	3_Architect	0.217	0.147	0.070	0.019	3.760**	.002	.578
1_Neighbour	3_Architect	0.232	0.147	0.085	0.019	4.596***	.001	.706
2_Developer	3_Architect	0.215	0.147	0.068	0.019	3.664**	.003	.563

Supplementary Table 4. The result of Tukey-HSD test for Actors. * $p < .05$, ** $p < 0.01$, *** $p < 0.001$

The post-hoc Tukey test on Actors showed that actor pairs 0-3, 1-3, and 2-3 were significantly different $p < .005$ while the difference between other actor pairs were insignificant. This leads to the conclusion that actors 0,1, and 2 were similar, actor 3 was quite different and actor 4 was lying somewhere in the middle.

The post-hoc Tukey test on the Sites showed that the none of the sites were treated significantly different by the decisions. This entails that the treatment of all sites by actors in their decision making is not meaningfully different in this test case. It can be conjectured that the relative unfamiliarity of the actors with the district renders their decisions monotonous w.r.t. the sites.

A	B	mean(A)	mean(B)	diff	*SE*	*T*	*p*-tukey	hedges
0_Reidential	1_Commercial	0.139	0.193	-0.055	0.016	-3.394**	.004	-.467
0_Reidential	2_Cultural	0.139	0.229	-0.090	0.016	-5.608***	.001	-.771
0_Reidential	3_Public	0.139	0.241	-0.102	0.016	-6.346***	.001	-.873
1_Commercial	3_Public	0.193	0.241	-0.048	0.016	-2.952*	.018	-.406

Supplementary Table 5. The result of Tukey-HSD test for Colors. * $p < .05$, ** $p < 0.01$, *** $p < 0.001$

The post-hoc Tukey test on Colors showed that color pairs 0-1, 0-2, and 0-3 were significantly different with $p < .005$, color pair 1-3 had also a relatively significant difference with $p < .05$, while the difference between other color pairs were insignificant. This entails that the most pairs of colored resources have been treated differently by the actors.

Decision Change

In this hypothesis-testing procedure, we want to check whether the decisions of the players are changing [significantly] as the game progresses. Therefore we formulate a hypothesis as "Decisions are changing throughout the rounds." Note that for this change to convey a sense of progress, we need to have a guage of convergence or a single metric, as to which we could consider the weighted product of all aggregate quality criteria as a single score. While this sense of progress is shown in the game dashboard already to all players, the one discussed here is merely about the ANOVA in between the axes of rounds of the game and the average colour percentages over these rounds, as the main objects of interest in the game illustrating the categorical investments. Note that there is already a given district level distribution of colours that we refer to as a programme of requirements which is being used as the basis for the iterative proportional fitting of all proposed colours to site allocations. These colour distributions could eventually also be compared to that PoR. However, since this PoR was not explained to the players extensively we cannot expect to make a meaningful conclusion from such a comparison in this case.

Round	mean	standard deviation
0	0.201	0.109
1	0.196	0.131
2	0.203	0.127

Supplementary Table 6. Mean and Standard Deviation over the decisions of each round.

Parameter	Value
Test statistics (W)	0.467
Degrees of freedom (df)	11
p value	.141

Supplementary Table 7. The result of levene to check the assumption of ANOVA

The data-set with two-way design of round-color has equal variance with Levene test ($W = 1.467$, $df = 11$, $p = .1412$). Normal ANOVA is used for later comparison.

Source	SS	df	MS	F	p	η^2
Round	0.004	2	0.002	0.131	.877	.001
Color	0.665	3	0.222	16.241	.000	.107
Round * Color	0.108	6	0.018	1.321	.246	.019
Residual	5.573	408	0.014	NaN	NaN	NaN

Supplementary Table 8. The result of two-way ANOVA test for the final workshop decisions across rounds; assessing the effect interaction of Round and Color

The two-way ANOVA shows that the main effects of Round is not significant: $F_{\mathrm{r}}(2,408) = 0.131$, $p > .05, \eta^2 = .001$; the main effect of Color is significant: $F_{\mathrm{c}}(3,408) = 16.241$, $p < .001, \eta^2 = .107$. The interaction effect between color and round is not significant: $F_{\mathrm{r*c}}(6,408) = 1.321$, $p > .05, \eta^2 = .019$.

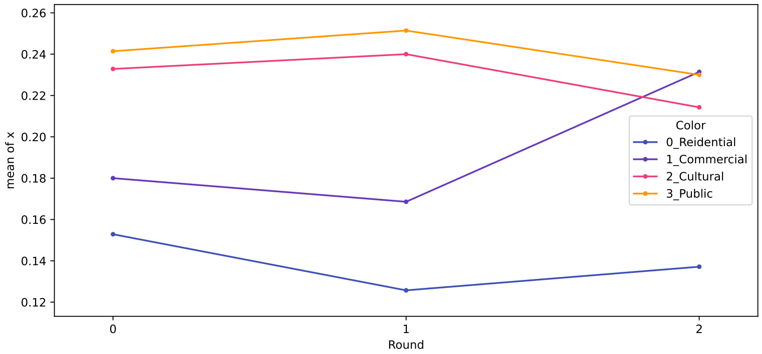

Supplementary Figure 9. Interaction plot of rounds and colours, revealing that the average percentages of colours allocated to the whole district change radically at least in the last round, with a considerably higher percentage of commercial space allocations

The interaction patterns of Round-Color (see Figure 9) shows that different colors are distributed equally for each round, showing that no significant changes have occurred along the game-play rounds. These insignificant variations in how spaces in each site are allocated to each color, are more visible in Figure 10.

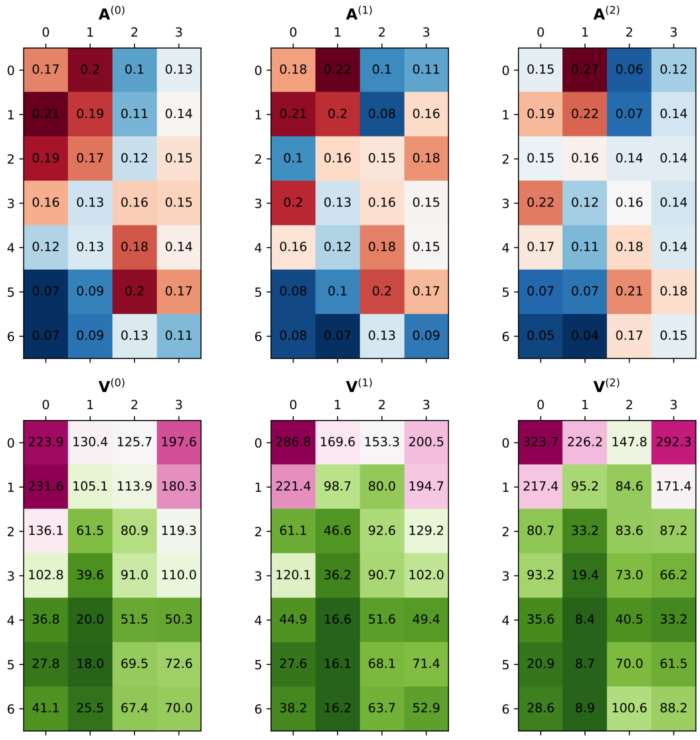

Supplementary Figure 10. Tensor plots of $\mathbf{A}^{(t)}_{n\times o}$ and $\mathbf{V}^{(t)}_{n\times o}$ during the three rounds of the final workshop, revealing that there exists some level of coherence amongst the decision tensors throughout the rounds and yet the tensors are obviously pointing into different directions, hence the exploratory relevance of the game

Discussion Time

Finally we looked into whether longer discussions in the rounds could yield a significantly higher score. To check this we propose to compute the Pearson correlation between the time spent in each round and the difference in the score of the round compared to previous round. In this test we consider all of the group scores ($\mathbf{q}$) and individual scores (gains). Nonetheless, since in the final workshop the players have only played three rounds, there would be only two samples for the correlation. Therefore, for this case we refer to Figure 11 to conclude that the difference between the scores of the first and second round is negligible in most of the scores except closeness score. Subsequently, the scores in round three have dropped compared to the second round.

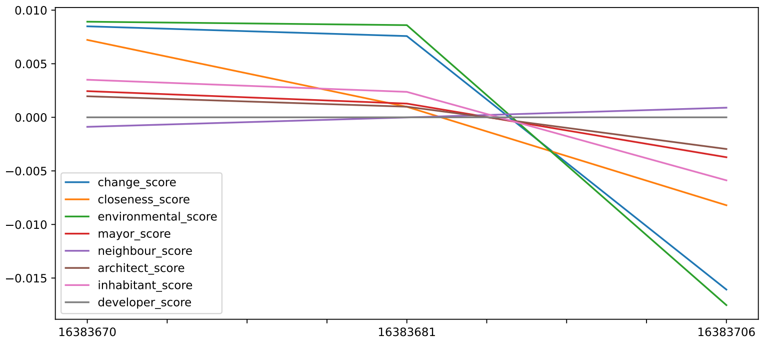

Supplementary Figure 11. group and individual scores at the end of rounds, revealing that the neighbour and the developer are the only actors who have consistently improved their gains as to their initial agenda cards; while the aggregate sustainability scores have dropped in the last round, calling for further attention to the complexity of the goal-oriented spatial design

Data Availability

The datasets generated and/or analysed during the current study are available in the EquiCityData repository https://github.com/shervinazadi/EquiCity_Data.

Author contributions statement

Contributions stated according to the CRediT – Contributor Roles Taxonomy:

PZN: Conceptualization, Methodology, Formal analysis, Software, Visualization, Writing- original draft preparation, Funding acquisition, Investigation, Supervision, Project administration, Validation. **SAZ**: Conceptualization, Methodology, Formal Analysis, Software, Data curation, Visualization, Funding Acquisition, Resources, Writing – review & editing. **NBI**: Validation, Formal analysis, Investigation, Writing – review & editing. **BDA**: Conceptualization, Data curation, Investigation. **NAZ**: Software, Resources. **SRZ**: Conceptualization, Writing- original draft preparation **APR**: Conceptualization, Supervision, Funding acquisition. All authors reviewed the manuscript.

References

1. Golub, G. H. & Van Loan, C. F. *Matrix computations* (JHU press, 2013).

2. Batty, M. Evolving a Plan: Design and Planning with Complexity. In Portugali, J. & Stolk, E. (eds.) *Complexity, Cognition, Urban Planning and Design*, Springer Proceedings in Complexity, 21–42, DOI: 10.1007/978-3-319-32653-5_2 (Springer International Publishing, Cham, 2016).

3. Friedkin, N. E., Proskurnikov, A. V., Mei, W. & Bullo, F. Mathematical Structures in Group Decision-Making on Resource Allocation Distributions. *Sci. Reports* **9**, 1377, DOI: 10.1038/s41598-018-37847-2 (2019). Bandiera_abtest: a Cc_license_type: cc_by Cg_type: Nature Research Journals Number: 1 Primary_atype: Research Publisher: Nature Publishing Group Subject_term: Applied mathematics;Mechanical engineering Subject_term_id: applied-mathematics;mechanical-engineering.

4. Batty, M. A Theory of Markovian Design Machines. *Environ. Plan. B: Plan. Des.* DOI: 10.1068/b010125 (1974).

5. Friedkin, N. E. *A Structural Theory of Social Influence* (Cambridge University Press, 1998), 1 edn.

6. Stephan, F. F. An iterative method of adjusting sample frequency tables when expected marginal totals are known. *The Annals Math. Stat.* **13**, 166–178 (1942). Publisher: JSTOR.

7. Deming, W. E. & Stephan, F. F. On a Least Squares Adjustment of a Sampled Frequency Table When the Expected Marginal Totals are Known. *The Annals Math. Stat.* **11**, 427–444, DOI: 10.1214/aoms/1177731829 (1940).

8. Hunsinger, E. Iterative Proportional Fitting For A Two-Dimensional Table (2008).

9. Simon, H. A. *Administrative behavior* (Simon and Schuster, 1997).

10. Jackson, M. O. Mechanism Theory. SSRN Scholarly Paper ID 2542983, Social Science Research Network, Rochester, NY (2014). DOI: 10.2139/ssrn.2542983.

11. Hwang, C.-L. & Lin, M.-J. *Group Decision Making under Multiple Criteria*, vol. 281 of *Lecture Notes in Economics and Mathematical Systems* (Springer Berlin Heidelberg, Berlin, Heidelberg, 1987).

12. Steunenberg, B., Schmidtchen, D. & Koboldt, C. Strategic Power in the European Union: Evaluating the Distribution of Power in Policy Games. *J. Theor. Polit.* **11**, 339–366, DOI: 10.1177/0951692899011003005 (1999). Publisher: SAGE Publications Ltd.

13. Nourian, P. *Configraphics: Graph Theoretical Methods for Design and Analysis of Spatial Configurations* (TU Delft Open, 2016).
